# Dengue hospitalizations in Brazil: Forecasting with climatic and physicians’ digital search data under real-world reporting delays

**DOI:** 10.1371/journal.pdig.0001206

**Published:** 2026-05-29

**Authors:** Dayanna Quintanilha Palmer, Marcela Motta, Eduardo Moura, Danielly Xavier, Guilherme Schittine, Angélica Caseri, Ronaldo Gismondi

**Affiliations:** 1 Department of Clinical Medicine, Universidade Federal Fluminense (UFF), Niterói, Rio de Janeiro, Brazil; 2 Research and Innovation Center, Afya, São Paulo, Brazil; 3 Institute of Mathematical and Computer Sciences (ICMC), Universidade de São Paulo (USP), São Paulo, Brazil; University of Washington, UNITED STATES OF AMERICA

## Abstract

Timely forecasting of dengue hospitalizations is essential for public health preparedness but is frequently limited by delays in official reporting systems. While climatic variables are known to influence dengue transmission and can be obtained in near-real time, hospitalization data often become available only weeks after patient admission, reducing their value for early response. Digital information generated during clinical practice, such as physicians’ search patterns, may provide a complementary and more timely signal of emerging disease activity. This study evaluates whether integrating climate data with real-time records of physicians’ searches for dengue-related information improves short-term forecasts of dengue hospitalizations in Brazil under both ideal and realistic reporting conditions. Three complementary data sources were combined to generate forecasts across multiple geographic regions: weekly hospitalization counts, climatic indicators, and anonymized physician search records from a widely used clinical decision-support platform. Model performance was compared under two scenarios: one assuming immediate availability of hospitalization data and another incorporating typical reporting delays. When hospitalization data were timely, simpler model configurations — particularly those relying on hospitalization history alone or combined with climate — achieved the highest predictive accuracy, indicating that the temporal structure of the outcome itself carried substantial forecasting value. Under realistic reporting delays, however, models incorporating physicians’ search behavior consistently outperformed all other approaches across most regions. In several regions, increases in physician search activity preceded or coincided with rises in hospital admissions, indicating early clinical engagement with dengue cases. These findings indicate that physician search behavior constitutes a valuable real-time indicator of dengue activity. Integrating digital clinical behavior with climate data enhances forecasting performance under real-world reporting constraints and may strengthen early-warning systems and public health decision-making for dengue and other climate-sensitive diseases.

## Author summary

Dengue is a major public health challenge in Brazil, where large outbreaks place sudden pressure on health services. Although climate conditions influence dengue transmission, public health responses often rely on hospitalization data that become available only weeks or months after patients are admitted, limiting the ability to act early. In this study, we explored whether real-time digital information generated by physicians could help overcome this delay. We combined weather data with anonymized records of physicians’ searches for dengue-related information within a widely used clinical decision-support platform in Brazil. We then tested whether these digital signals could improve short-term forecasts of dengue hospitalizations across different regions of the country, especially when official hospital data were delayed. We found that when hospitalization data were available without delays, simpler models based on hospitalization history and climate patterns performed well. However, under realistic reporting delays, models that incorporated physicians’ search behavior produced more accurate forecasts across most regions. These findings show that digital clinical behavior can provide early insight into rising disease activity and support more timely public health responses.

## 1. Introduction

Dengue virus (DENV) is an arbovirus with four distinct serotypes (DENV-1 to DENV-4), primarily transmitted by *Aedes* mosquitoes, especially *Aedes aegypti*. While primary infection may be asymptomatic or present with mild symptoms, severe cases can progress to dengue hemorrhagic fever and dengue shock syndrome, characterized by coagulopathy and increased vascular permeability, which can ultimately be fatal if not promptly recognized and managed [1,2]. In 2024, Brazil recorded approximately 6.42 million probable dengue cases, representing a 327% increase compared to 2023, when 1.51 million probable cases were reported [3].

Several factors may contribute to the rise in dengue cases, with climatic conditions playing a central role in mosquito-borne disease dynamics. Temperatures exceeding regional threshold levels accelerate the life cycle of dengue vectors (*Aedes aegypti*, and *Aedes albopictus*), increase dengue virus proliferation and mosquito bite frequency, and shorten the extrinsic incubation period [4,5]. Higher humidity and precipitation have been linked to increased dengue transmission, as they create favorable conditions for virus replication and spread [4,6]. Furthermore, socio-economic factors play an important role in shaping dengue transmission patterns, contributing to a complex system that influences the probability of future outbreaks [7,8].

Accurately mapping geographic distribution and predicting outbreaks is essential to guide resource allocation and enable timely public health interventions [6,9]. Several predictive models have been proposed, but most focus exclusively on climate variables [8]. In addition, forecasting efforts are also constrained by reporting delays in official hospitalization and case-notification systems, which reduce the timeliness and operational usefulness of surveillance data [10]. Moreover, digital behavioral data from physicians—such as clinical search activity within medical decision-support platforms—have not yet been explored in the context of dengue modeling in Brazil, despite their potential to provide early indicators of clinical demand and contribute to real time decision making.

Integrating meteorological and non-climatic factors may substantially improve predictive accuracy [11,12]. In this context, digital health data provides a novel opportunity for syndromic surveillance [13,14]. Patterns of access to medical applications can reflect real-time shifts in medical concerns and population health trends. In Brazil, the Afya Whitebook application, a widely used digital health tool among physicians, captures search behaviors, including queries related to dengue. In a previous study, these digital traces showed significant correlation with hospitalizations for dengue, supporting their use as a proxy of clinical demand [15].

Building upon this rationale, the present study proposes a predictive framework to forecast weekly dengue hospitalizations in Brazil by integrating climatic variables, modeled with lagged predictors to capture delayed environmental effects, and digital health indicators derived from physicians’ search behavior within the Afya Whitebook platform. To address hospitalization reporting delays, we developed two modeling scenarios: an ideal-data model, assuming real-time data availability, and a real-world model, which incorporates the typical delay observed in hospitalization record updates. By comparing these model configurations, we aim to evaluate how incorporating realistic data latency and temporal dependencies can enhance the accuracy and operational relevance of short-term dengue forecasting.

## 2. Methods

### 2.1. Ethics statement

The research complies with the ethical principles established by Resolution CNS 466/12 of the Brazilian National Health Council. All data used were anonymized and derived from public or internal databases, with no individual-level identifiers or direct participant interaction.

The research protocol was reviewed and approved by the Research Ethics Committee of the Centro Universitário Presidente Tancredo de Almeida Neves (UNIPTAN), under CAAE 81123624.4.0000.9667 and Opinion number 6.944.607. The Committee granted a waiver of informed consent given the exclusive use of anonymized secondary data.

### 2.2. Study design

**Fig 1:**
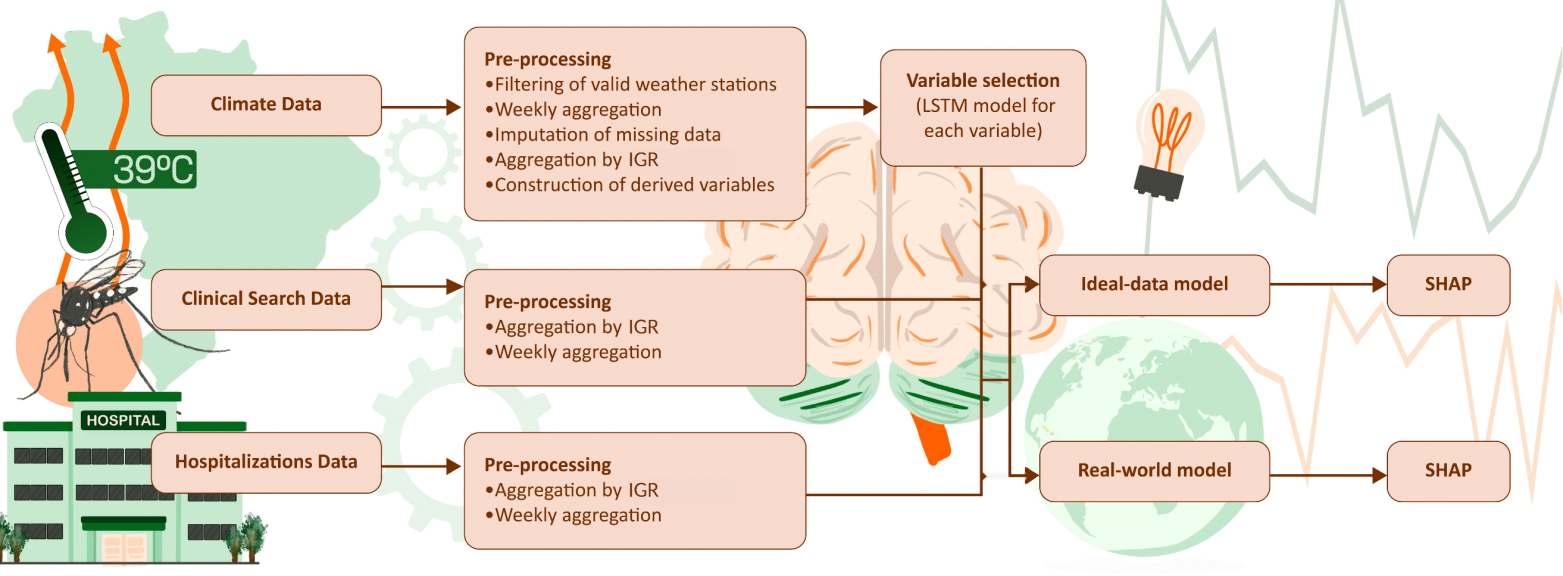
Workflow of the analytical pipeline, including preprocessing of weather station data, weekly aggregation, imputation of missing values, IGR-level aggregation, derivation of climate variables, LSTM-based variable selection, and SHAP analyses for both ideal-data and real-world scenarios.

We developed a forecasting framework based on Long Short-Term Memory (LSTM) neural networks to predict dengue-related hospitalizations in Brazil at the Immediate Geographic Region (IGR) level. **Fig 1** provides an overview of the analytical workflow, which includes:

(i) integration of climate, physicians` clinical search, and hospitalization datasets from public service;
(ii) preprocessing steps such as weekly aggregation, imputation of missing values, and consolidation at the IGR level;
(iii) derivation of additional climate indicators;
(iv) variable screening using LSTM-based relevance evaluation; and
(v) model training and interpretability using SHAP (SHapley Additive Explanations) values.

Given the temporal nature of the data and the need to capture both short and long-term dependencies in dengue transmission, we employed LSTM networks, a type of recurrent neural network specifically designed to handle sequential data with temporal correlations [16]. LSTM models have been successfully applied in infectious disease forecasting, including dengue [11], demonstrating strong performance in predicting case trends based on climatic and other exogenous variables.

The modeling strategy was designed to generate 8-week-ahead forecasts, a horizon chosen to reflect both epidemiological relevance and operational usefulness for public health planning. To capture the intrinsic temporal structure of dengue transmission, we implemented a rolling sliding-window approach, in which recent historical sequences were used to predict subsequent hospitalization counts. This setup enabled the model to learn meaningful temporal patterns and produce actionable short- to medium-term forecasts.

### 2.3. Study site

According to the Brazilian Institute of Geography and Statistics (IBGE), an IGR corresponds to a group of neighboring municipalities organized around one or more urban centers that function as local hubs for goods, services, and labor. These regions reflect short-distance functional relationships, particularly commuting flows and the daily circulation of people and economic activities.

The current territorial division, comprises 510 IGRs, that represent an intermediate geographic scale between municipalities and states, ensuring spatial coherence for socioeconomic, environmental, and health analyses [17].

### 2.4. Data sources

#### 2.4.1. Hospitalizations data.

The SIH/SUS database, maintained by the Department of Informatics of the Unified Health System (DATASUS), provides anonymized, publicly available information on hospital admissions throughout Brazil [18]. Data are updated monthly, with a typical release delay of one to two months after hospital care. In accordance with Portaria SAES nº 1.110/2021 of the Brazilian Ministry of Health, the final consolidation of hospitalization data within the SIH/SUS may occur up to four months after the reference month, due to the administrative cycles of data submission, verification, and correction of hospitalization authorizations [10].

#### 2.4.2. Clinical search data.

Afya Whitebook is a widely used clinical decision-support platform among healthcare professionals in Brazil. The platform provides structured, evidence-based content such as disease overviews, diagnostic algorithms, therapeutic guidelines, drug dosing information, and clinical calculators. Optimized for mobile and desktop use, it supports real-time clinical decision-making across a variety of care settings. Although its primary audience is licensed physicians, the user base also includes residents, medical students, and other allied health professionals. For this study, only search data generated by verified licensed physicians with active professional credentials were included. In Brazil, there were 597,428 practicing physicians as of January 2024, according to the recent edition of Demografia Médica [19]. In comparison, the Afya Whitebook platform registered approximately 150,000 monthly active physician users by the end of the same year [20].

However, user density varies considerably across regions, with mean annual active physicians per IGR in the study sample ranging from approximately 200 (Pirapora) to over 68,000 (São Paulo) (S1 Table). This heterogeneity is addressed by training independent models per IGR and is further discussed in the Limitations. The Afya Whitebook research database contains metadata on user search behavior, collected in real time and accessible retrospectively for research purposes via a structured query interface. All data are anonymized to ensure confidentiality and compliance with ethical standards, preventing the identification of individual healthcare professionals.

#### 2.4.3. Climate data.

Daily observations of precipitation, maximum, mean, and minimum temperature, and mean relative humidity were obtained from the Brazilian National Institute of Meteorology (Instituto Nacional de Meteorologia – INMET) automatic weather stations distributed throughout Brazil [21].

### 2.5. Data pre-processing

**Fig 2:**
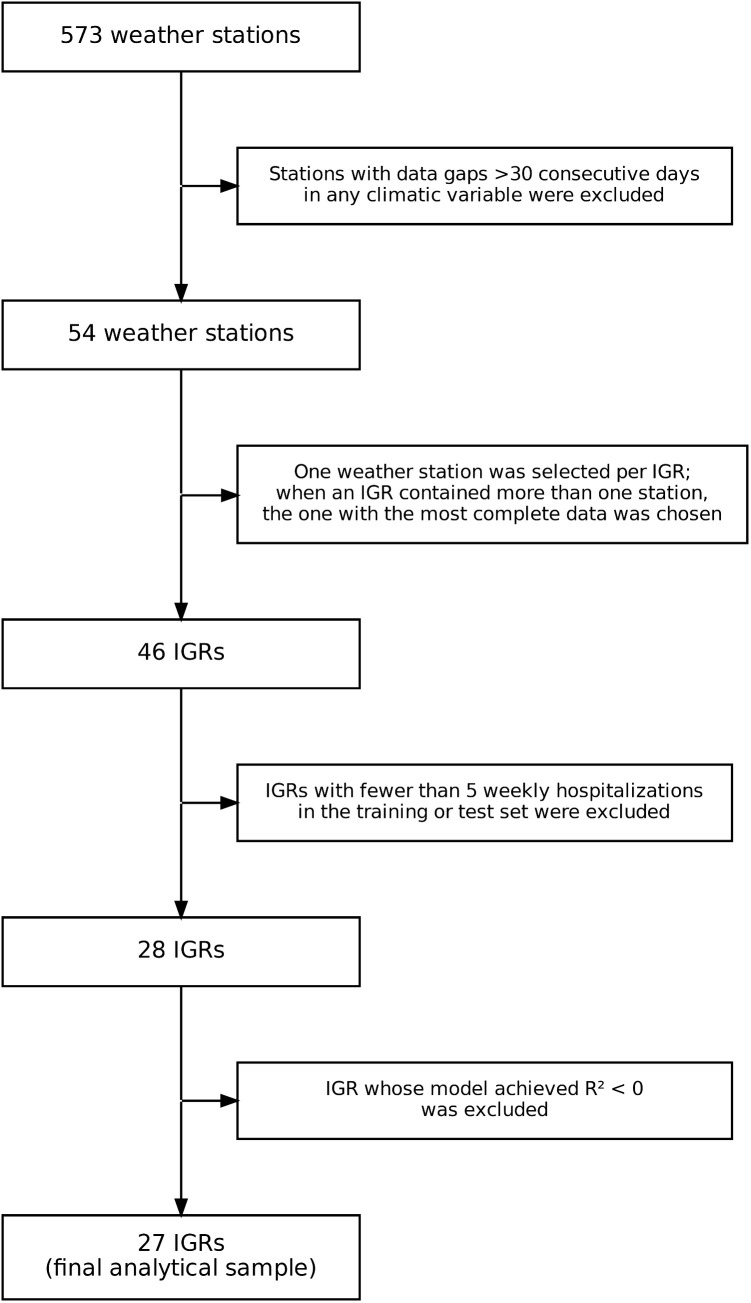
Flowchart of weather station and Immediate Geographic Region (IGR) selection. From the 573 INMET weather stations initially available, sequential exclusions were applied based on climatic data completeness (>30 consecutive missing days), one-station-per-IGR selection criteria, minimum weekly hospitalization counts in the training and test sets (≥5), and model performance (R² ≥ 0). The final analytical sample comprised 27 IGRs. INMET, Instituto Nacional de Meteorologia; IGR, Immediate Geographic Region; R², coefficient of determination.

Climate data underwent cleaning and harmonization: dates were converted to datetime format, variable names standardized, and station metadata (name, geographic coordinates, operational period) appended to each record. Only observations between 2021 and 2024 were retained. The selection of analytical units followed the sequential criteria summarized in **Fig 2**. Of the 573 INMET weather stations operating during the study period, 54 met the completeness threshold (no gaps exceeding 30 consecutive days in any climatic variable).

For these 54 stations, daily measures were aggregated into weekly series — averages for temperature and humidity, and weekly sums for precipitation. Missing weekly values were imputed using a centered moving average computed with two weeks forward and two weeks backward (minimum of one available neighbor), ensuring temporal continuity of the time series. Each Immediate Geographic Region (IGR) was then represented by a single station; when more than one eligible station fell within the same IGR, the one with the highest percentage of complete observations was retained, yielding 46 IGRs.

These 46 IGRs were cross-referenced with dengue hospitalization records from the SIH/SUS system (510 IGRs with reported hospitalizations between 2021 and 2024) and with clinical search data from the Afya Whitebook platform. IGRs with fewer than 5 weekly hospitalizations in either the training or test set were excluded, reducing the sample to 28 IGRs. After preliminary modeling, one additional IGR was removed because its baseline LSTM model produced a non-positive R², indicating insufficient temporal structure for forecasting.

The final analytical sample comprised 27 IGRs: Alegre, Belo Horizonte, Campina Grande, Campos dos Goytacazes, Catalão, Cruz Alta, Distrito Federal, Frederico Westphalen, Ijuí, Juiz de Fora, Linhares, Marília, Maringá, Oliveira, Passo Fundo, Passos, Pirapora, Porto Alegre, Ribeirão Preto, Rio de Janeiro, Salvador, Santa Cruz do Sul, Santa Maria, São Miguel do Oeste, São Paulo, Uberaba, and Uberlândia. These regions presented sufficient data density across all domains to enable robust multi-step forecasting.

### 2.6. Study variables

The primary outcome of interest was the weekly number of hospitalizations for dengue fever extracted from the SIH/SUS database. Predictor variables encompassed both meteorological and digital health domains. Meteorological predictors included lagged indicators of precipitation, temperature, and humidity (1–4 weeks), alongside a structured derivation process that resulted in a final set of 42 climate-related variables, including weekly change flags, interaction terms, extreme climate indicators, seasonal dummies, temperature categories, and precipitation occurrence and patterns. In addition, clinical search data were incorporated through weekly counts of physician searches for dengue in Afya Whitebook, restricted to verified licensed physicians. Together, these variables were selected to capture the combined influence of climatic fluctuations and real-time medical information-seeking behavior on dengue-related hospitalizations. All derived variables are summarized in Table 1, and their detailed definitions and computation procedures are provided in the Supplementary Material (S2 Table).

**Table 1:** Summary of candidate predictors and variable categories considered for inclusion in the models.

Category	Variable Type	Concise Description	Representative Unit(s)
Temperature	Mean, Lagged, Quartiles, Change, Extremes	Weekly mean temperature, lagged values (1–4 weeks), quartile indicators, and binary indicators for percentile changes and extremes (≥95th percentile).	°C, Binary (0/1)
Precipitation	Sum, Count, Lagged, Change, Extremes	Weekly total precipitation, rainy-day count, lagged values (1–4 weeks), and binary indicators for weekly percentile change and extremes (≥95th percentile).	mm, Count (days), Binary (0/1)
Humidity	Mean, Lagged, Change, Extremes	Weekly mean relative humidity, lagged values (1–4 weeks), and binary indicators for percentile changes and extremes (≥95th percentile).	%, Binary (0/1)
Season	Seasonal Indicators	Binary indicators for the four climatic seasons (Summer, Autumn, Winter, Spring).	Binary (0/1)
Clinical Access	Normalized search rate	Weekly number of clinical searches performed by physicians on the Afya Whitebook platform, normalized by the number of active physicians in the IGR in that year.	Searches per 10,000 active physicians

Source: Climate data were obtained from INMET; clinical search data were obtained from Afya Whitebook. Full construction rules are provided in S2 Table (supplement).

### 2.7. Feature selection

Given the presence of 42 climatic input variables derivations, we sought to reduce model dimensionality and avoid multicollinearity by selecting only one variable from each climatic category—humidity, temperature, precipitation, and seasonality. To achieve this, we employed a variable-by-variable evaluation approach, in which the model was trained using the hospitalization variable alongside a single climatic predictor at a time. For each category, the climatic variable yielding the lowest RMSE was retained. As a result, each IGR retained four climatic input variables, one from each category. This strategy was intended to reduce redundancy among correlated climatic predictors before model fitting and to improve the interpretability of subsequent SHAP analyses. Feature importance was further assessed using SHAP values, which enabled interpretation of the most influential predictors per IGR and quantification of the relative contribution of climatic versus digital health variables.

### 2.8. Data analysis

Hospitalization, digital clinical search, and climatic datasets were merged at the IGR–week level to form a unified analytic panel. Exploratory analyses included visualization of temporal trends and descriptive statistics across regions. Forecasting was performed using a LSTM neural network trained on weekly climatic and digital health predictors.

Forecasting was performed using a LSTM neural network trained on weekly climatic and digital health predictors. The complete dataset spans 2021–2024, totaling 208 epidemiological weeks. Because the model relies on lagged predictors (1–4 weeks), the first 4 weeks were discarded, leaving 204 usable weeks (SE 5/2021 to SE 52/2024). These were split chronologically in a 70/30 ratio: the training set comprised the first 143 weeks (SE 5/2021 to SE 43/2023, late October 2023), and the holdout test set comprised the remaining 61 weeks (SE 44/2023 to SE 52/2024).

Input–output pairs were constructed using a sliding-window approach: a window of 24 consecutive weeks served as the model input, and the immediately following 8 weeks as the prediction target. The window advanced one week at a time along the time series, generating successive overlapping input–output pairs. This procedure was applied independently to the training and test sets. The model was trained exclusively on the training set, and the test set was reserved solely for performance evaluation. All reported results therefore correspond to forecasts on data the model never saw during training.

All data preprocessing and modeling were conducted in a Python-based analytical environment [22]. Hyperparameters were optimized using a grid search evaluating different architectural configurations, including the number of hidden layers (1–4) and hidden units (20 or 40). The final model employed an input window of 24 weeks (seq_len = 24), a prediction horizon of 8 weeks (output_size = 8), a learning rate of 0.01, and a MSE loss function. Each configuration was trained in triplicate to account for stochastic variability during optimization. Model performance was assessed using the RMSE, the MSE, the Mean Absolute Error (MAE), and the coefficient of determination (R²).

These metrics were computed as follows:


$$RMSE=\sqrt{\left(\left(\text{1}/n\right)\cdot \Sigma \left({\left(y_{t}-{\hat{y}}_{t}\right)}^{\text{2}}\right)\right)}$$

$$MSE=\left(\text{1}/n\right)\cdot \Sigma \left({\left(y_{t}-{\hat{y}}_{t}\right)}^{\text{2}}\right)$$

$$MAE=\left(\text{1}/n\right)\cdot \Sigma \left|\;y_{t}-{\hat{y}}_{t}\;\right|$$

To compute the coefficient of determination, we used the Residual Sum of Squares (RSS) and the Total Sum of Squares (TSS):


$$RSS=\Sigma \left({\left(y_{t}-{\hat{y}}_{t}\right)}^{\text{2}}\right)$$

$$TSS=\Sigma \left({\left(y_{t}-\bar{y}\right)}^{\text{2}}\right)$$

$${\text{R}}^{\text{2}}=\text{1}-(\text{RSS}/\text{TSS})$$

These quantities quantify both the magnitude of prediction errors and the proportion of variance in weekly hospitalizations explained by the forecasting model.

To evaluate the relative contribution of different data domains, five model structures were compared:

- **Hospitalization-only model (LSTM: Hospitalization):** Trained exclusively on past hospitalization counts to capture the intrinsic temporal dynamics of dengue without external predictors.
- **Clinical-search model (LSTM: Hospitalization + Clinical Search):** Trained using hospitalization time series combined with Afya Whitebook physician search data.
- **Climate model (LSTM: Hospitalization + Climate):** Trained using hospitalization data and climatic predictors, selecting the best-performing variable from each domain (temperature, humidity, precipitation, season).
- **Integrated model (LSTM: Hospitalization + Clinical Search + Climate):** Combines hospitalization data, climatic indicators, and real-time physician search behavior, restricted to the most informative variable per domain.
- **SARIMAX model (SARIMAX: Hospitalization):** A classical benchmark trained only on hospitalization counts, used as a baseline for comparative performance assessment [23].

In addition, two complementary versions were implemented:

- **Ideal-data model:** predictors and hospitalization data were temporally aligned.
- **Real-world model:** hospitalization data were shifted by up to eight weeks (approximately two months).

The two-month delay window was empirically defined based on the average reporting lag observed in the hospitalization dataset, ensuring that the model reflected the actual latency pattern present in official records. Pairwise differences in RMSE between models were assessed across the triplicate runs of each scenario. Normality of the paired differences was first evaluated using the Shapiro–Wilk test; when normality was supported, a paired t-test was applied, and the Wilcoxon signed-rank test was used otherwise. Statistical significance was reported at p < 0.05, with additional thresholds at p < 0.01 and p < 0.001.

Model adequacy was assessed through residual diagnostics. The Autocorrelation Function (ACF) of model residuals was evaluated up to lag 8 to verify whether residuals behaved as white noise (autocorrelations within ±1.96/√n). The Ljung-Box test (α = 0.05) was applied to formally test the absence of serial autocorrelation in the residuals. The Time-Lagged Cross-Correlation (TLCC) between predicted and observed series was computed to assess temporal alignment, with a satisfactory fit defined by a peak correlation at lag 0.

#### 2.8.1. LSTM model architecture.

LSTM networks are a special type of recurrent neural network (RNN) designed to overcome the vanishing and exploding gradient problem in long sequences [16]. An LSTM unit contains a memory cell with a constant error carousel (CEC) that preserves information across time steps, while multiplicative gates regulate information flow. Gers, Schmidhuber, and Cummins (2000) extended the model with the forget gate, allowing adaptive resetting of memory contents [24].

In our study, the LSTM architecture was adapted to model the temporal dynamics of dengue hospitalizations in Brazil. The input, forget, and output gates controlled the flow of information across time, while the memory cell preserved long-range dependencies, allowing the model to integrate both climatic fluctuations and digital health signals. Following this principle, we structured the implementation to use weekly lagged climate indicators, extreme event markers, and physician search behavior from Afya Whitebook as inputs. Through a sliding-window training strategy, the LSTM was able to capture short-term variations and longer seasonal patterns, providing forecasts at the IGR level that aligned with the theoretical design of the architecture.

## 3. Results

### 3.1. Spatiotemporal trends in hospitalization and clinical search data (2021–2024)

**Table 2** presents dengue hospitalization counts and rates across IGRs for the 2021–2024 period. Smaller-population regions such as Frederico Westphalen, Oliveira, São Miguel do Oeste, and Uberlândia exhibited high hospitalization rates, frequently surpassing 200–600 per 100,000 inhabitants in their peak year, while large metropolitan centers, including São Paulo and Rio de Janeiro, reported low hospitalization rates despite contributing some of the highest absolute numbers.

**Table 2:** Hospitalizations and rates per 100000 inhabitants in 27 selected Brazilian Immediate Geographic Regions, 2021–2024.

IGRs	Population	2021		2022		2023		2024	
		*N*	*Rate*	*N*	*Rate*	*N*	*Rate*	*N*	*Rate*
Alegre	193,922	4	2.1	19	9.8	166	85.6	245	126.3
Belo Horizonte	5,045,254	46	0.9	117	2.3	996	19.7	8,119	160.9
Campina Grande	938,502	129	13.7	275	29.3	50	5.3	319	34.0
Campos dos Goytacazes	631,164	4	0.6	27	4.3	321	50.9	726	115.0
Catalão	170,345	45	26.4	215	126.2	36	21.1	279	163.8
Cruz Alta	138,860	1	0.7	4	2.9	81	58.3	112	80.7
Distrito Federal	2,817,381	411	14.6	1,292	45.9	1,005	35.7	7,408	262.9
Frederico Westphalen	119,099	32	26.9	228	191.4	18	15.1	742	623.0
Ijuí	203,825	2	1.0	27	13.2	55	27.0	373	183.0
Juiz de Fora	711,921	9	1.3	17	2.4	122	17.1	1,052	147.8
Linhares	333,129	62	18.6	27	8.1	174	52.2	288	86.5
Marília	380,834	78	20.5	182	47.8	110	28.9	245	64.3
Maringá	811,915	43	5.3	477	58.8	278	34.2	950	117.0
Oliveira	115,926	1	0.9	62	53.5	93	80.2	469	404.6
Passo Fundo	278,694	3	1.1	7	2.5	49	17.6	108	38.8
Passos	258,889	3	1.2	97	37.5	349	134.8	671	259.2
Pirapora	133,939	1	0.7	11	8.2	146	109.0	296	221.0
Porto Alegre	2,997,642	6	0.2	595	19.8	310	10.3	1,619	54.0
Ribeirão Preto	1,446,059	19	1.3	191	13.2	260	18.0	1,101	76.1
Rio de Janeiro	11,760,550	21	0.2	151	1.3	1,061	9.0	3,095	26.3
Salvador	3,485,014	11	0.3	167	4.8	749	21.5	369	10.6
Santa Cruz do Sul	364,672	80	21.9	27	7.4	12	3.3	149	40.9
Santa Maria	461,725	1	0.2	4	0.9	130	28.2	157	34.0
São Miguel do Oeste	172,124	11	6.4	533	309.7	34	19.8	406	235.9
São Paulo	20,731,920	125	0.6	141	0.7	207	1.0	7,436	35.9
Uberaba	454,392	19	4.2	106	23.3	231	50.8	206	45.3
Uberlândia	961,202	42	4.4	456	47.4	1,404	146.1	2,902	301.9

**Source:** Hospitalization data from the Brazilian Hospital Information System (SIH/DATASUS); population estimates from IBGE. N = annual number of dengue-related hospitalizations. Rate = (N ÷ IGR population) × 100,000.

Despite regional variability, most IGRs exhibited a progressive increase in hospitalizations over the study period, with the most pronounced growth occurring between 2023 and 2024. However, several IGRs deviated from this general upward trend, particularly between 2022 and 2023. São Miguel do Oeste experienced the most pronounced decline, dropping from a rate of 309.7 to 19.8 per 100,000—a reduction of over 90%—following one of the highest hospitalization rates observed across all IGRs during the study period. Similar post-peak declines were observed in Frederico Westphalen (191.4 to 15.1 per 100,000), Catalão (126.2 to 21.1 per 100,000), and Campina Grande (29.3 to 5.3 per 100,000). All four regions subsequently rebounded in 2024. Santa Cruz do Sul displayed a distinct non-linear pattern, with an early peak in 2021, a progressive decline through 2023 (21.9 to 3.3 per 100,000), and a renewed increase in 2024. Other regions, such as Passo Fundo and Santa Maria, began with very low hospitalization rates in 2021–2022 but rose to moderate levels by 2023–2024, reflecting a delayed but evident escalation.

As shown in **Table 3**, most IGRs displayed higher dengue-related search activity in the later years of the study period compared with 2021. Although the trajectory was not strictly linear, many regions showed a general upward tendency over time, with notable increases in 2022 and again in 2024. This temporal pattern is consistent with the national hospitalization profile, which also registered higher counts in these two years.

**Table 3:** Dengue-related searches and access rates per 10000 physicians in 27 Brazilian IGRs, 2021–2024.

IGRs	2021		2022		2023		2024	
	N	Rate	N	Rate	N	Rate	N	Rate
Alegre	10	324	44	1,232	56	1,436	96	2,866
Belo Horizonte	2,296	716	5,502	1,651	9,302	4,102	11,802	5,127
Campina Grande	176	742	277	1,234	102	426	289	1,903
Campos dos Goytacazes	22	218	56	549	90	1,266	119	1,288
Catalão	14	560	69	2,491	37	1,085	139	3,861
Cruz Alta	12	531	65	2,234	97	2,948	149	3,296
Distrito Federal	2,586	2,324	4,927	3,675	3,153	2,594	4,808	3,883
Frederico Westphalen	15	630	89	3,739	47	1,506	199	7,210
Ijuí	27	572	147	2,442	206	3,527	297	5,756
Juiz de Fora	91	351	147	572	309	964	711	3,468
Linhares	47	641	53	566	147	1,557	197	3,031
Maringá	172	635	664	1,733	501	1,582	753	2,454
Marília	65	617	186	1,662	188	1,366	325	1,822
Oliveira	18	418	71	1,170	140	2,541	129	2,198
Passo Fundo	30	174	158	1,808	229	2,700	283	3,406
Passos	10	220	116	1,629	171	2,627	198	3,225
Pirapora	6	392	33	1,803	228	10,411	81	3,389
Porto Alegre	930	606	5,181	2,486	3,171	2,483	5,421	3,534
Ribeirão Preto	189	308	442	799	500	1,113	1,028	1,988
Rio de Janeiro	2,653	656	5,161	1,309	8,161	2,422	11,130	2,927
Salvador	1,335	1,111	2,635	2,207	2,860	2,362	3,882	3,275
Santa Cruz do Sul	83	797	184	1,785	107	1,269	276	3,977
Santa Maria	61	550	158	1,352	282	2,730	580	5,390
São Miguel do Oeste	11	420	92	2,636	71	2,407	137	4,774
São Paulo	8,556	1,167	11,669	1,652	11,012	1,712	28,384	4,228
Uberaba	64	339	260	1,527	310	2,248	355	2,647
Uberlândia	146	474	620	1,990	965	2,855	1,075	3,161

Source: Afya Whitebook Access and Engagement Data. Internal company platform. N = number of dengue-related searches. Rate = (N ÷ number of physicians active on the Afya Whitebook platform in the corresponding year) × 10000. Annual physician counts per IGR are provided in S1 Table.

### 3.2. Correlation analysis between hospitalizations, climate variables, and digital clinical search activity

**Fig 3:**
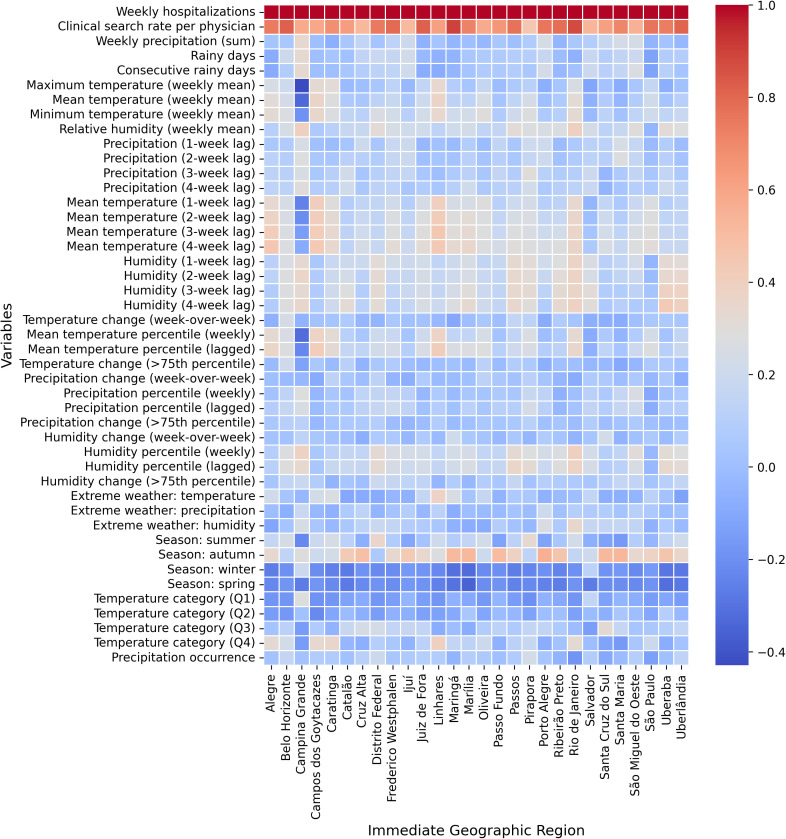
Correlation matrix showing pairwise correlations between dengue hospitalizations, Afya Whitebook search activity, and climate-related predictors across the 27 Immediate Geographic Regions, computed prior to feature selection. Afya Whitebook access demonstrates the strongest association with hospitalizations.

The resulting correlation matrix revealed a clear contrast in the strength and consistency of associations across variable categories, as illustrated in **Fig 3**. Afya Whitebook access demonstrated the strongest and most frequent positive correlation with dengue hospitalizations across IGRs.

In contrast, climatic predictors displayed predominantly weak and heterogeneous correlations with dengue hospitalizations across IGRs. Humidity and temperature indicators showed low to moderate associations, with no consistent pattern across regions. Precipitation-related variables exhibited minimal and highly variable correlations, without any clear or recurrent signal in most regions.

**Fig 4:**
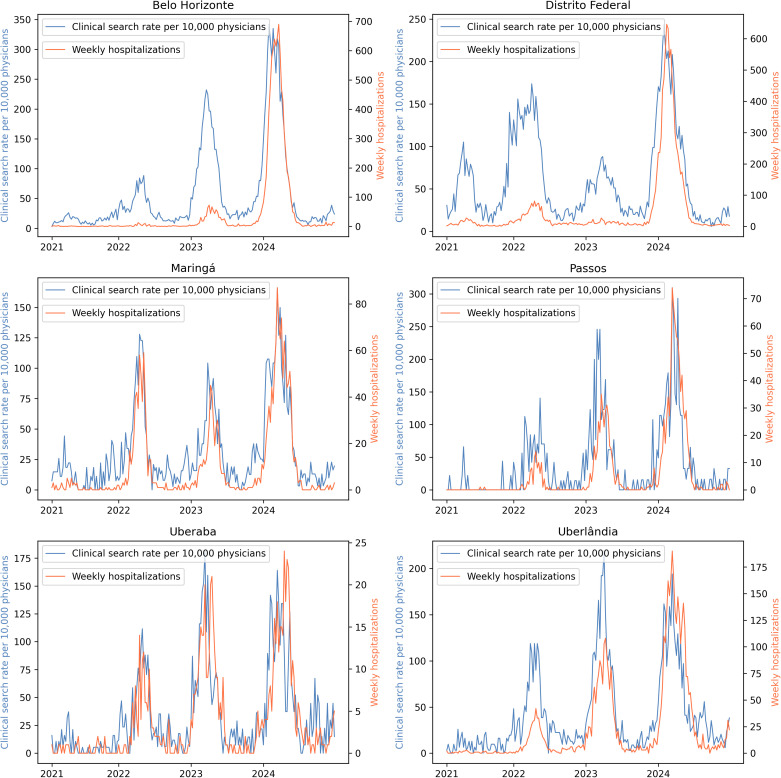
Weekly dengue hospitalizations (orange line) and Afya Whitebook dengue-related search rate per 10,000 active physicians (blue line) across Immediate Geographic Regions from 2021 to 2024. Six of the 27 IGRs were selected as representative examples for visualization. Peaks in clinical search activity typically precede or coincide with hospitalization increases, indicating early clinical engagement with dengue and supporting its use as a real-time digital indicator.

Time-series curves of Afya Whitebook search rates and dengue hospitalizations further supported these findings, as illustrated in **Fig 4**. In nearly all IGRs, peaks in dengue-related search activity preceded or coincided with increases in hospitalizations.

### 3.3. Selected variables across immediate geographic regions (IGRs)

During the feature-selection procedure, we ensured that each IGR retained at least one predictor from the four predefined climatic categories—humidity, temperature, precipitation, and seasonality—reflecting their established relevance in dengue transmission dynamics. Despite this harmonized structure, the LSTM model selected markedly different climatic signatures across regions. Some IGRs were primarily influenced by short-term humidity fluctuations or temperature lags, whereas others showed stronger contributions from precipitation extremes, or accumulated rainfall.

The full set of predictors selected for each IGR, grouped by climatic category, is provided in S3 Table.

### 3.4. Predictive performance of climate, digital, and integrated models

To improve visual interpretability, we highlighted 12 selected Immediate Geographic Regions (IGRs) representing distinct forecasting contexts: major urban centers with high epidemiological relevance, regions with clearer gains from clinical-search data, regions with marked performance shifts after reporting delays were introduced, and atypical cases illustrating geographic heterogeneity. The full set of other 15 IGRs is presented in S1 Fig.

**Fig 5:**
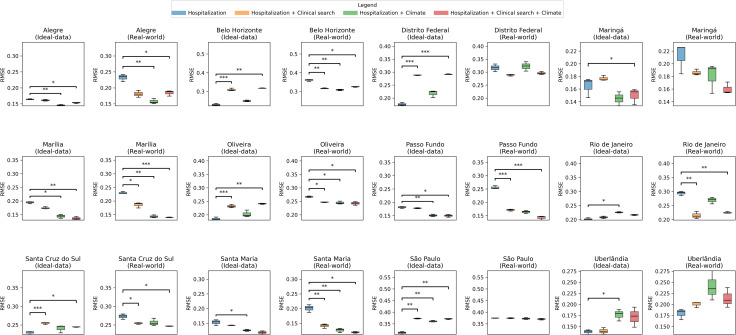
Distribution of root mean squared error (RMSE) across triplicate model runs, by Immediate Geographic Region (IGR), scenario, and feature set. Boxplots show the distribution of RMSE values obtained from three independent training runs of the LSTM model for each of the 12 IGRs included in the study, under two evaluation scenarios: Ideal-data (left panel of each IGR pair) and Real-world (right panel). For each scenario, four feature-set configurations were compared: hospitalizations only (blue), hospitalizations + clinical search data (orange), hospitalizations + climatic variables (green), and hospitalizations + clinical search + climatic variables (red). Boxes represent the interquartile range (IQR), horizontal lines indicate the median, whiskers extend to the minimum and maximum values within 1.5 × IQR, and dots denote outliers. Brackets and asterisks above each panel indicate statistically significant pairwise differences between the hospitalizations-only baseline and the feature-augmented models, based on a paired t-test (when paired differences were normally distributed by the Shapiro–Wilk test) or a Wilcoxon signed-rank test (otherwise) (* p < 0.05, ** p < 0.01, *** p < 0.001). Overall, the incorporation of clinical search data and/or climatic variables reduced RMSE relative to the hospitalizations-only baseline in most IGRs, with the magnitude and direction of improvement varying across regions and between the ideal-data and real-world scenarios.

Under the ideal-data scenario (**Fig 5**, left panels), simpler model configurations — particularly the Hospitalization-only and Climate models — more often achieved the lowest prediction errors, suggesting that when hospitalization records are available without reporting delays, the temporal structure of the outcome variable itself provides substantial predictive information.

After introducing real-world reporting delays (**Fig 5**, right panels), the relative ranking of models shifted. Models incorporating clinical-search data — either alone or combined with climate — more frequently emerged among the best-performing approaches, indicating that real-time physician search behavior helped compensate for the reduced temporal informativeness of delayed hospitalization records.

Taken together, these results indicate that the predictive value of each data domain depended on data availability conditions. Simpler models performed well when hospitalization data were timely, whereas models incorporating physician digital behavior became more advantageous under real-world reporting delays — the scenario most representative of operational public health conditions. Detailed RMSE values (mean ± standard error) and pairwise statistical comparisons for all 27 IGRs are provided in S4–S7 Tables.

### 3.5. Model diagnostics

Across the 216 LSTM model fits (four configurations × 27 IGRs × two scenarios), residual diagnostics confirmed adequate model behavior. ACF analysis showed that residuals fell within the white-noise band (±1.96/√n) for all 216 series, indicating that systematic temporal patterns were captured by the models. The Ljung-Box test corroborated this finding, with all p-values > 0.05, supporting the absence of serial autocorrelation. The TLCC analysis revealed peak correlation at lag 0 — indicating no systematic temporal shift between predicted and observed series — in 92 of 108 Ideal-data models (85%) and 79 of 108 Real-world models (73%). The slightly lower proportion under real-world conditions is consistent with the eight-week reporting delay incorporated in this scenario.

### 3.6. SHAP

**Fig 6:**
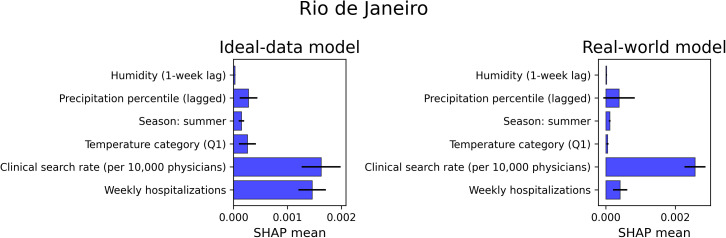
SHAP feature importance for the Rio de Janeiro IGR under Ideal-data and Real-world LSTM scenarios. Mean absolute SHAP values for the six predictors of the integrated model. The Ideal-data model (left) assumes temporal alignment between predictors and hospitalization counts; the Real-world model (right) incorporates an eight-week reporting lag in hospitalization data.

When comparing SHAP patterns across scenarios (**Fig 6**; Supplementary S2 Fig and S3 Fig), a consistent shift emerged: the predictive influence of hospitalization counts decreased markedly in the Real-world model, reflecting the loss of temporal alignment introduced by reporting delays. As hospitalization features became less informative, clinical-search indicators gained prominence, emerging as the most influential domain under delayed conditions and effectively compensating for the degraded hospitalization signal. Climatic predictors retained moderate relevance across all scenarios, though their contribution varied substantially across regions—some IGRs were driven by humidity-related changes, others by temperature categories or precipitation lags—highlighting strong geographic heterogeneity in the role of environmental covariates.

## 4. Discussion

This study yielded three main findings. First, dengue-related searches on the Afya Whitebook platform showed strong temporal alignment with hospitalizations, with search peaks often preceding or coinciding with increases in admissions. Second, climatic predictors varied substantially across IGRs, reflecting regional heterogeneity in environmental drivers. Third, under realistic reporting delays, models incorporating physician digital search behavior — either alone or combined with climate — outperformed approaches relying solely on hospitalization history or climate information, indicating that physician search behavior provides timely epidemiological information that compensates for the loss of predictive signal caused by delayed hospitalization data.

Between 2021 and 2024, clinical searches and hospitalizations increased across regions, with strong temporal alignment between the two. This indicates that physicians rapidly adjust their information-seeking behavior in response to changes in clinical demand, consistent with evidence that digital engagement can serve as an early indicator of infectious disease activity [13,14]. Prior studies have shown that public digital indicators—such as Twitter activity—can forecast dengue trends by several weeks [25]. By using physician search data rather than public-facing digital behavior, our study likely provides a more precise and clinically relevant digital signal, directly connected to medical decision-making and more suitable for operational forecasting.

However, not all IGRs followed this general upward trajectory. Several regions, most notably São Miguel do Oeste, Frederico Westphalen, and Catalão, showed marked declines in hospitalizations after high-incidence years, a pattern that may reflect temporary depletion of susceptible individuals following intense local transmission. In São Miguel do Oeste, for example, the hospitalization rate peaked at 309.7 per 100,000 inhabitants in 2022 and dropped sharply in 2023. This interpretation is consistent with previous studies showing that the size of successive dengue waves can be shaped by the interaction between herd immunity and seasonal transmission [26]. Changes in local climatic dynamics may also have contributed to these temporal fluctuations. These regional deviations further underscore the importance of accounting for local dynamics [11], consistent with our modeling strategy.

Climatic variables contributed mainly through lagged effects, aligning with findings from Chen & Moraga (2025) that forecasting accuracy improves when models explicitly incorporate temporal delays in environmental exposures [11] In our study, contemporaneous associations between climate and hospitalizations were modest, but the LSTM consistently selected lagged temperature, humidity, and precipitation indicators as relevant inputs, and SHAP analyses confirmed their predictive influence. These results reinforce that climate is a key determinant of dengue dynamics, but its effects become detectable only when temporal structure and non-linear relationships are modeled explicitly.

Model comparisons further supported these patterns. Under ideal-data conditions, simpler configurations — particularly the Hospitalization-only model — most frequently achieved the lowest prediction errors, indicating that when hospitalization records are available without delays, their temporal structure alone carries substantial forecasting value. Climate-based models also performed well in a subset of regions, consistent with the recognized environmental dependence of dengue transmission [4,5]. When realistic reporting delays were introduced, this pattern shifted markedly: models incorporating clinical-search data dominated, achieving the best performance in the majority of regions. Digital search data gained importance by providing real-time information that compensated for the diminished value of delayed hospitalization counts, while climatic predictors retained a complementary role.

SHAP analyses clarified the interplay between predictors across scenarios. In the absence of reporting delays, climatic variables and recent hospitalization history dominated model contributions, whereas under delayed-reporting conditions, the influence of hospitalization history decreased sharply and digital search activity rose in relative importance. Together, these findings demonstrate that medium-range climatic signals and real-time behavioral indicators provide complementary information, enhancing the accuracy and operational value of forecasting models. This has practical implications for environmental and health surveillance: in settings where reporting delays are structural, incorporating digital search behavior into forecasting frameworks can improve situational awareness, support earlier public health responses, and strengthen regional early-warning systems. Similar benefits have been observed in other hybrid environmental–digital approaches used for dengue nowcasting in Brazil [27], reinforcing the potential of integrating clinical digital signals into surveillance tools.

Several limitations should be considered when interpreting the generalizability of our findings. First, because the analysis was based on aggregated data at the IGR level rather than individual patient records, the models were designed to predict regional hospitalization counts and cannot be used to estimate individual-level risk of dengue. Accordingly, the ecological design restricts inference to population-level associations and precludes causal interpretation at the individual level.

Second, SIH/SUS hospitalization data may be affected by underreporting and reporting delays, potentially distorting temporal dengue patterns. In addition, hospitalization records capture only the most severe cases requiring inpatient care, whereas mild or asymptomatic infections—which account for most dengue cases—are not represented. The proportion of dengue cases progressing to hospitalization may also vary across IGRs due to differences in healthcare access, distance to facilities, and regional clinical practices, which may lead to underestimation of disease burden in some settings. However, because each IGR was modeled independently, the LSTM was able to learn region-specific relationships between predictors and local hospitalization patterns.

Third, uneven coverage of INMET meteorological stations led to the exclusion of regions with substantial data gaps, limiting nationwide applicability. Moreover, each IGR was represented by a single weather station selected on the basis of data completeness, which may not fully capture climatic heterogeneity within larger or geographically diverse regions.

Fourth, Afya Whitebook search data, although high-volume, represent only a subset of clinicians and were unevenly distributed across regions, with mean annual active users per IGR ranging from approximately 200 in smaller regions to more than 68,000 in large urban centers. This likely reflects an urban–rural gradient in digital platform adoption. Although the analysis was restricted to verified physician accounts, these data may still overrepresent younger, urban, or more digitally engaged clinicians. In addition, concurrent outbreaks of other febrile illnesses with overlapping clinical presentations, such as chikungunya or Zika, may have increased dengue-related searches as part of the differential diagnosis, potentially inflating the clinical-search signal independently of true dengue activity. Although dengue was the dominant arboviral disease in Brazil during the study period, this remains a possible source of confounding.

Fifth, the absence of entomological or virological indicators, such as vector density or circulating serotypes, may have limited the model’s sensitivity to short-term transmission shifts. In addition, although SHAP analyses improved interpretability, LSTM models remain only partially interpretable. Hyperparameter tuning was also performed using the test set, because the limited length of the time series (204 usable weeks) precluded the creation of a separate validation partition without substantially reducing the training data. This strategy may have introduced optimistic bias in absolute performance estimates, although it is less likely to have affected the relative ranking of models, since all architectures were subjected to the same selection procedure. Future studies based on longer time series should adopt dedicated validation sets or nested cross-validation strategies.

Finally, several authors are affiliated with Afya, the company that owns the clinical platform evaluated in this study. Although these relationships were fully disclosed and Afya had no role in the study design, analysis, interpretation, manuscript preparation, or decision to publish, this context should be considered when interpreting the findings.

To reduce these potential sources of bias, we restricted Afya Whitebook data to verified physician accounts, applied standardized cleaning procedures to the SIH/SUS time series, and limited the analysis to IGRs with adequate climatic data coverage. In addition, results were interpreted at the IGR level, where temporal aggregation helps reduce random fluctuations and reporting noise. Nevertheless, these precautions do not eliminate residual bias, and the findings should be interpreted in light of these limitations.

This study demonstrates that clinician search behavior within a point-of care medical decision tool platform can serve as a timely and reliable digital indicator of dengue activity in Brazil. By integrating climatic conditions with real-time physician search data into our models, we consistently improve the forecasting of dengue hospitalizations, particularly when accounting for reporting delays. This integrated approach provides a more rapid and clinically grounded signal to support outbreak detection and public health decision-making. The next steps are to assess this framework’s generalizability across diverse settings and then evaluate its suitability for operational early-warning use, paving the way for its real-world application.

## Supporting information

S1 Table Number of physicians with access to the Afya Whitebook platform per IGR and year 2021–2024. (DOCX)

S2 Table Climatic and clinical variables used in the forecasting models. Description of all climatic, seasonal, and physician clinical-search variables, including units, derivation, and lagged indicators.
(DOCX)

S3 Table Predictors selected across Immediate Geographic Regions. Climatic and digital predictors selected by the machine learning model across all Immediate Geographic Regions.
(DOCX)

S4 Table Model performance under ideal-data conditions. Root mean squared error values across regions for all predictive model configurations.
(DOCX)

S5 Table Statistical comparison of models under ideal-data conditions. Pairwise statistical tests comparing predictive performance.
(DOCX)

S6 Table Model performance under real-world reporting conditions. Root mean squared error values across regions accounting for reporting delays.
(DOCX)

S7 Table Statistical comparison of models under real-world reporting conditions. Pairwise statistical tests comparing predictive performance under reporting delays.
(DOCX)

S8 Table Model diagnostics under ideal-data conditions. (CSV)

S9 Table Model diagnostics under Real-world conditions (eight-week reporting delay). (CSV)

S1 Fig Distribution of root mean squared error (RMSE) across triplicate model runs — complementary set of Immediate Geographic Regions (IGRs). (TIF)

S2 Fig SHAP-based feature importance across all 27 IGRs under the Ideal-data scenarios. (TIF)

S3 Fig SHAP-based feature importance across all 27 IGRs under the Real-world scenarios. (TIF)
