# Supplementary material for: Dengue hospitalizations in Brazil: Forecasting with climatic and physicians’ digital search data under real-world reporting delays
Source: PLOS Digit Health. 2026 May 29;5(5):e0001206. doi: 10.1371/journal.pdig.0001206 (PMC13221015; doi:10.1371/journal.pdig.0001206)
Supplement: S1 Table — (DOCX) [file pdig.0001206.s001.docx]

**S1 Table. Annual count of physicians active on the Afya Whitebook platform per Immediate Geographic Region (IGR), Brazil, 2021–2024.**

| Immediate Geographic Region | 2021 | 2022 | 2023 | 2024 |
| --- | --- | --- | --- | --- |
| Alegre | 309 | 357 | 390 | 335 |
| Belo Horizonte | 32,062 | 33,319 | 22,675 | 23,019 |
| Campina Grande | 2,373 | 2,245 | 2,397 | 1,519 |
| Campos dos Goytacazes | 1,009 | 1,020 | 711 | 924 |
| Catalão | 250 | 277 | 341 | 360 |
| Cruz Alta | 226 | 291 | 329 | 452 |
| Distrito Federal | 11,128 | 13,408 | 12,157 | 12,381 |
| Frederico Westphalen | 238 | 238 | 312 | 276 |
| Ijuí | 472 | 602 | 584 | 516 |
| Juiz de Fora | 2,594 | 2,568 | 3,206 | 2,050 |
| Linhares | 733 | 937 | 944 | 650 |
| Maringá | 2,708 | 3,831 | 3,167 | 3,069 |
| Marília | 1,054 | 1,119 | 1,376 | 1,784 |
| Oliveira | 431 | 607 | 551 | 587 |
| Passo Fundo | 1,722 | 874 | 848 | 831 |
| Passos | 455 | 712 | 651 | 614 |
| Pirapora | 153 | 183 | 219 | 239 |
| Porto Alegre | 15,348 | 20,837 | 12,769 | 15,338 |
| Ribeirão Preto | 6,135 | 5,529 | 4,491 | 5,172 |
| Rio de Janeiro | 40,419 | 39,424 | 33,698 | 38,026 |
| Salvador | 12,015 | 11,937 | 12,106 | 11,852 |
| Santa Cruz do Sul | 1,042 | 1,031 | 843 | 694 |
| Santa Maria | 1,110 | 1,169 | 1,033 | 1,076 |
| São Miguel do Oeste | 262 | 349 | 295 | 287 |
| São Paulo | 73,318 | 70,656 | 64,323 | 67,129 |
| Uberaba | 1,886 | 1,703 | 1,379 | 1,341 |
| Uberlândia | 3,083 | 3,115 | 3,380 | 3,401 |

***Source:*** *Afya Whitebook Access and Engagement Data (internal company platform). Counts represent the number of unique physicians who accessed the platform at least once during each calendar year in the corresponding IGR. These values were used as denominators to compute normalized search rates per 10,000 physicians in Table 3.*
