# Supplementary material for: Dengue hospitalizations in Brazil: Forecasting with climatic and physicians’ digital search data under real-world reporting delays
Source: PLOS Digit Health. 2026 May 29;5(5):e0001206. doi: 10.1371/journal.pdig.0001206 (PMC13221015; doi:10.1371/journal.pdig.0001206)
Supplement: S2 Table — Description of all climatic, seasonal, and physician clinical-search variables, including units, derivation, and lagged indicators. (DOCX) [file pdig.0001206.s002.docx]

**S2 Table. Climatic and clinical variables used in the models.**

| Variable name | Description | Type / Unit | Source / Derivation |
| --- | --- | --- | --- |
| PHYSICIANS_CLINICAL_SEARCH | Weekly count of physicians' clinical searches | Count (searches) | Whitebook clinical search logs |
| PRECIPITATION_WEEKLY_SUM | Weekly sum of precipitation | mm (weekly sum) | Daily weather measurements |
| RAINY_DAYS | Number of rainy days in the week | Count (days) | Daily precipitation measurements |
| TEMPERATURE_MEAN_WEEKLY_MAX | Weekly mean of daily maximum temperatures | °C (weekly mean) | Daily weather measurements |
| TEMPERATURE_MEAN_WEEKLY_MEAN | Weekly mean of daily mean temperature | °C (weekly mean) | Daily weather measurements |
| TEMPERATURE_MEAN_WEEKLY_MIN | Weekly mean of daily minimum temperatures | °C (weekly mean) | Daily weather measurements |
| HUMIDITY_MEAN_WEEKLY | Weekly mean of daily relative humidity | % (weekly mean) | Daily weather measurements |
| LAG_1_PRECIPITATION | Precipitation one week before | mm (weekly sum) | Daily weather measurements (lagged) |
| LAG_2_PRECIPITATION | Precipitation two weeks before | mm (weekly sum) | Daily weather measurements (lagged) |
| LAG_3_PRECIPITATION | Precipitation three weeks before | mm (weekly sum) | Daily weather measurements (lagged) |
| LAG_4_PRECIPITATION | Precipitation four weeks before | mm (weekly sum) | Daily weather measurements (lagged) |
| LAG_1_TEMPERATURE_MEAN | Mean temperature one week before | °C (weekly mean) | Daily weather measurements (lagged) |
| LAG_2_TEMPERATURE_MEAN | Mean temperature two weeks before | °C (weekly mean) | Daily weather measurements (lagged) |
| LAG_3_TEMPERATURE_MEAN | Mean temperature three weeks before | °C (weekly mean) | Daily weather measurements (lagged) |
| LAG_4_TEMPERATURE_MEAN | Mean temperature four weeks before | °C (weekly mean) | Daily weather measurements (lagged) |
| LAG_1_HUMIDITY | Mean humidity one week before | % (weekly mean) | Daily weather measurements (lagged) |
| LAG_2_HUMIDITY | Mean humidity two weeks before | % (weekly mean) | Daily weather measurements (lagged) |
| LAG_3_HUMIDITY | Mean humidity three weeks before | % (weekly mean) | Daily weather measurements (lagged) |
| LAG_4_HUMIDITY | Mean humidity four weeks before | % (weekly mean) | Daily weather measurements (lagged) |
| TEMPERATURE_CHANGE | Weekly change in temperature percentile· Temperature percentile moves into the 0·75–0·85 range from a value below 0·75 | Binary (0/1) | Weekly comparison with previous distribution |
| PERCENTILE_TEMPERATURE_MEAN_WEEKLY_MEAN | Percentile of weekly mean temperature | — | Weekly distribution processing |
| PERCENTILE_LAG_TEMPERATURE_MEAN_WEEKLY_MEAN | Percentile of lagged weekly mean temperature | — | Weekly distribution processing |
| CHANGE_TEMPERATURE_GREATER75 | Weekly mean change in temperature percentile when it rises from below 0·75 to 0·75 or higher | Binary (0/1) | Weekly comparison with previous distribution |
| CHANGE_PRECIPITATION | Weekly change in precipitation percentile· Precipitation percentile moves into the 0·75–0·85 range from a value below 0·75 | Binary (0/1) | Weekly comparison with previous distribution |
| PERCENTILE_PRECIPITATION_WEEKLY_SUM | Percentile of weekly precipitation sum | — | Weekly distribution processing |
| PERCENTILE_LAG_PRECIPITATION_WEEKLY_SUM | Percentile of lagged weekly precipitation sum | — | Weekly distribution processing |
| CHANGE_PRECIPITATION_GREATER75 | Precipitation percentile rises from below 0·75 to 0·75 or higher | Binary (0/1) | Weekly comparison with previous distribution |
| CHANGE_HUMIDITY | Weekly change in humidity percentile· Humidity percentile moves into the 0·75–0·85 range from a value below 0·75 | Binary (0/1) | Weekly comparison with previous distribution |
| PERCENTILE_HUMIDITY_MEAN_WEEKLY | Percentile of weekly mean humidity | — | Weekly distribution processing |
| PERCENTILE_LAG_HUMIDITY_MEAN_WEEKLY | Percentile of lagged weekly mean humidity | — | Weekly distribution processing |
| CHANGE_HUMIDITY_GREATER75 | Humidity percentile rises from below 0·75 to 0·75 or higher | Binary (0/1) | Weekly comparison with previous distribution |
| EXTREME_CLIMATE_TEMPERATURE | Weekly mean temperature above 95th percentile | Binary (0/1) | Weekly extreme thresholds |
| EXTREME_CLIMATE_PRECIPITATION | Weekly sum precipitation above 95th percentile | Binary (0/1) | Weekly extreme thresholds |
| EXTREME_CLIMATE_HUMIDITY | Weekly mean humidity above 95th percentile | Binary (0/1) | Weekly extreme thresholds |
| SEASON_SUMMER | Summer indicator | Binary (0/1) | Brazilian climatological calendar |
| SEASON_AUTUMN | Autumn indicator | Binary (0/1) | Brazilian climatological calendar |
| SEASON_WINTER | Winter indicator | Binary (0/1) | Brazilian climatological calendar |
| SEASON_SPRING | Spring indicator | Binary (0/1) | Brazilian climatological calendar |
| CATEGORY_TEMPERATURE_QUANTILE_01 | Temperature in the 0–25th percentile range | Binary (0/1) | Weekly distribution processing |
| CATEGORY_TEMPERATURE_QUANTILE_02 | Temperature in the 26–50th percentile range | Binary (0/1) | Weekly distribution processing |
| CATEGORY_TEMPERATURE_QUANTILE_03 | Temperature in the 51–75th percentile range | Binary (0/1) | Weekly distribution processing |
| CATEGORY_TEMPERATURE_QUANTILE_04 | Temperature in the 76–100th percentile range | Binary (0/1) | Weekly distribution processing |
| PRECIPITATION_OCCURRENCE | Indicator of any precipitation during the week | Binary (0/1) | Daily precipitation measurements |
