# Supplementary material for: Dengue hospitalizations in Brazil: Forecasting with climatic and physicians’ digital search data under real-world reporting delays
Source: PLOS Digit Health. 2026 May 29;5(5):e0001206. doi: 10.1371/journal.pdig.0001206 (PMC13221015; doi:10.1371/journal.pdig.0001206)
Supplement: S3 Table — Climatic and digital predictors selected by the machine learning model across all Immediate Geographic Regions. (DOCX) [file pdig.0001206.s003.docx]

**S3 Table. Climatic features selected by the LSTM model, grouped by variable category across Immediate Geographic Regions (IGRs).**

| IGRs | Humidity | Temperature | Precipitation | Season |
| --- | --- | --- | --- | --- |
| Alegre | CHANGE_HUMIDITY_GREATER75 | LAG_1_TEMPERATURE_MEAN | PERCENTILE_LAG_WEEKLY_PRECIPITATION_SUM | SEASON_SUMMER |
| Belo Horizonte | PERCENTILE_HUMIDITY_MEAN_WEEKLY | LAG_2_TEMPERATURE_MEAN | LAG_4_PRECIPITATION | SEASON_SPRING |
| Campina Grande | LAG_3_HUMIDITY | CATEGORY_TEMPERATURE_QUANTILE_04 | PRECIPITATION_OCCURRENCE | SEASON_AUTUMN |
| Campos dos Goytacazes | HUMIDITY_MEAN_WEEKLY | CATEGORY_TEMPERATURE_QUANTILE_02 | LAG_1_PRECIPITATION | SEASON_SUMMER |
| Catalão | PERCENTILE_HUMIDITY_MEAN_WEEKLY | LAG_3_TEMPERATURE_MEAN | PRECIPITATION_OCCURRENCE | SEASON_SPRING |
| Cruz Alta | PERCENTILE_HUMIDITY_MEAN_WEEKLY | CHANGE_TEMPERATURE_GREATER75 | PRECIPITATION_OCCURRENCE | SEASON_SPRING |
| Distrito Federal | CHANGE_HUMIDITY | CHANGE_TEMPERATURE | LAG_4_PRECIPITATION | SEASON_SPRING |
| Frederico Westphalen | CHANGE_HUMIDITY_GREATER75 | CATEGORY_TEMPERATURE_QUANTILE_02 | EXTREME_CLIMATE_PRECIPITATION | SEASON_SPRING |
| Ijuí | EXTREME_CLIMATE_HUMIDITY | CHANGE_TEMPERATURE_GREATER75 | PERCENTILE_LAG_WEEKLY_PRECIPITATION_SUM | SEASON_AUTUMN |
| Juiz de Fora | EXTREME_CLIMATE_HUMIDITY | CATEGORY_TEMPERATURE_QUANTILE_04 | PRECIPITATION_OCCURRENCE | SEASON_WINTER |
| Linhares | HUMIDITY_MEAN_WEEKLY | CATEGORY_TEMPERATURE_QUANTILE_03 | LAG_4_PRECIPITATION | SEASON_SPRING |
| Maringá | CHANGE_HUMIDITY_GREATER75 | CATEGORY_TEMPERATURE_QUANTILE_02 | EXTREME_CLIMATE_PRECIPITATION | SEASON_SPRING |
| Marília | PERCENTILE_LAG_HUMIDITY_MEAN_WEEKLY | CATEGORY_TEMPERATURE_QUANTILE_01 | CHANGE_PRECIPITATION_GREATER75 | SEASON_AUTUMN |
| Oliveira | CHANGE_HUMIDITY_GREATER75 | TEMPERATURE_MEAN_WEEKLY_MEAN | RAINY_DAYS | SEASON_SPRING |
| Passo Fundo | LAG_4_HUMIDITY | PERCENTILE_WEEKLY_MEAN_TEMPERATURE | PERCENTILE_LAG_WEEKLY_PRECIPITATION_SUM | SEASON_SPRING |
| Passos | LAG_3_HUMIDITY | TEMPERATURE_MEAN_WEEKLY_MIN | RAINY_DAYS | SEASON_AUTUMN |
| Pirapora | LAG_3_HUMIDITY | CATEGORY_TEMPERATURE_QUANTILE_01 | LAG_1_PRECIPITATION | SEASON_AUTUMN |
| Porto Alegre | CHANGE_HUMIDITY | PERCENTILE_LAG_WEEKLY_MEAN_TEMPERATURE | PERCENTILE_LAG_WEEKLY_PRECIPITATION_SUM | SEASON_SPRING |
| Ribeirão Preto | CHANGE_HUMIDITY_GREATER75 | TEMPERATURE_MEAN_WEEKLY_MEAN | LAG_1_PRECIPITATION | SEASON_SPRING |
| Rio de Janeiro | LAG_1_HUMIDITY | CATEGORY_TEMPERATURE_QUANTILE_01 | PERCENTILE_LAG_WEEKLY_PRECIPITATION_SUM | SEASON_SUMMER |
| Salvador | LAG_2_HUMIDITY | CATEGORY_TEMPERATURE_QUANTILE_03 | PERCENTILE_WEEKLY_PRECIPITATION_SUM | SEASON_SUMMER |
| Santa Cruz do Sul | CHANGE_HUMIDITY | CHANGE_TEMPERATURE_GREATER75 | LAG_2_PRECIPITATION | SEASON_AUTUMN |
| Santa Maria | HUMIDITY_MEAN_WEEKLY | TEMPERATURE_MEAN_WEEKLY_MIN | LAG_4_PRECIPITATION | SEASON_SPRING |
| São Miguel do Oeste | HUMIDITY_MEAN_WEEKLY | PERCENTILE_WEEKLY_MEAN_TEMPERATURE | LAG_3_PRECIPITATION | SEASON_SPRING |
| São Paulo | EXTREME_CLIMATE_HUMIDITY | EXTREME_CLIMATE_TEMPERATURE | CHANGE_PRECIPITATION_GREATER75 | SEASON_WINTER |
| Uberaba | EXTREME_CLIMATE_HUMIDITY | CATEGORY_TEMPERATURE_QUANTILE_01 | PRECIPITATION_OCCURRENCE | SEASON_AUTUMN |
| Uberlândia | LAG_4_HUMIDITY | CATEGORY_TEMPERATURE_QUANTILE_04 | CHANGE_PRECIPITATION_GREATER75 | SEASON_WINTER |
