# Supplementary material for: Dengue hospitalizations in Brazil: Forecasting with climatic and physicians’ digital search data under real-world reporting delays
Source: PLOS Digit Health. 2026 May 29;5(5):e0001206. doi: 10.1371/journal.pdig.0001206 (PMC13221015; doi:10.1371/journal.pdig.0001206)
Supplement: S4 Table — Root mean squared error values across regions for all predictive model configurations. (DOCX) [file pdig.0001206.s004.docx]

**S4 Table: RMSE (mean ± SE) for five predictive models across 27 Immediate Geographic Regions under Ideal-data scenarios**

| Immediate Geographic Region | Sarima Univariate | Hospitalization | Hospitalization + Clinical search | Hospitalization + Climate | Hospitalization + Clinical search + Climate |
| --- | --- | --- | --- | --- | --- |
| Alegre | 0.171 ± 0.0 | 0.164 ± 0.001 | 0.161 ± 0.003 | 0.146 ± 0.002 | 0.153 ± 0.002 |
| Belo Horizonte | 0.341 ± 0.0 | 0.229 ± 0.006 | 0.31 ± 0.007 | 0.249 ± 0.004 | 0.317 ± 0.001 |
| Campina Grande | 0.235 ± 0.0 | 0.162 ± 0.001 | 0.165 ± 0.001 | 0.128 ± 0.001 | 0.135 ± 0.002 |
| Campos dos Goytacazes | 0.299 ± 0.0 | 0.252 ± 0.001 | 0.255 ± 0.032 | 0.273 ± 0.012 | 0.305 ± 0.012 |
| Catalão | 0.195 ± 0.0 | 0.172 ± 0.003 | 0.167 ± 0.001 | 0.157 ± 0.001 | 0.157 ± 0.005 |
| Cruz Alta | 0.139 ± 0.0 | 0.156 ± 0.01 | 0.161 ± 0.002 | 0.145 ± 0.004 | 0.134 ± 0.012 |
| Distrito Federal | 0.324 ± 0.0 | 0.176 ± 0.007 | 0.288 ± 0.001 | 0.218 ± 0.014 | 0.292 ± 0.001 |
| Frederico Westphalen | 0.181 ± 0.0 | 0.191 ± 0.002 | 0.152 ± 0.024 | 0.124 ± 0.015 | 0.158 ± 0.006 |
| Ijuí | 0.248 ± 0.0 | 0.233 ± 0.002 | 0.215 ± 0.004 | 0.194 ± 0.013 | 0.219 ± 0.007 |
| Juiz de Fora | 0.331 ± 0.0 | 0.279 ± 0.004 | 0.232 ± 0.023 | 0.252 ± 0.011 | 0.221 ± 0.006 |
| Linhares | 0.19 ± 0.0 | 0.187 ± 0.002 | 0.161 ± 0.007 | 0.198 ± 0.007 | 0.195 ± 0.014 |
| Maringá | 0.207 ± 0.0 | 0.165 ± 0.016 | 0.177 ± 0.004 | 0.145 ± 0.011 | 0.15 ± 0.013 |
| Marília | 0.243 ± 0.0 | 0.194 ± 0.004 | 0.174 ± 0.004 | 0.144 ± 0.007 | 0.136 ± 0.006 |
| Oliveira | 0.24 ± 0.0 | 0.185 ± 0.006 | 0.232 ± 0.006 | 0.203 ± 0.013 | 0.241 ± 0.002 |
| Passo Fundo | 0.173 ± 0.0 | 0.181 ± 0.004 | 0.178 ± 0.002 | 0.151 ± 0.004 | 0.15 ± 0.005 |
| Passos | 0.179 ± 0.0 | 0.128 ± 0.005 | 0.153 ± 0.008 | 0.166 ± 0.031 | 0.155 ± 0.001 |
| Pirapora | 0.204 ± 0.0 | 0.212 ± 0.006 | 0.214 ± 0.003 | 0.17 ± 0.015 | 0.185 ± 0.006 |
| Porto Alegre | 0.226 ± 0.0 | 0.202 ± 0.011 | 0.228 ± 0.001 | 0.188 ± 0.005 | 0.196 ± 0.031 |
| Ribeirão Preto | 0.291 ± 0.0 | 0.2 ± 0.006 | 0.22 ± 0.008 | 0.244 ± 0.011 | 0.24 ± 0.018 |
| Rio de Janeiro | 0.279 ± 0.0 | 0.202 ± 0.004 | 0.208 ± 0.004 | 0.226 ± 0.002 | 0.217 ± 0.002 |
| Salvador | 0.293 ± 0.0 | 0.104 ± 0.002 | 0.105 ± 0.003 | 0.115 ± 0.004 | 0.114 ± 0.003 |
| Santa Cruz do Sul | 0.266 ± 0.0 | 0.229 ± 0.004 | 0.255 ± 0.003 | 0.241 ± 0.01 | 0.245 ± 0.001 |
| Santa Maria | 0.116 ± 0.0 | 0.153 ± 0.01 | 0.143 ± 0.001 | 0.126 ± 0.003 | 0.118 ± 0.006 |
| São Miguel do Oeste | 0.118 ± 0.0 | 0.093 ± 0.002 | 0.089 ± 0.004 | 0.061 ± 0.002 | 0.06 ± 0.003 |
| São Paulo | 0.362 ± 0.0 | 0.311 ± 0.006 | 0.373 ± 0.002 | 0.361 ± 0.004 | 0.371 ± 0.003 |
| Uberaba | 0.15 ± 0.0 | 0.166 ± 0.012 | 0.143 ± 0.003 | 0.127 ± 0.004 | 0.13 ± 0.002 |
| Uberlândia | 0.214 ± 0.0 | 0.138 ± 0.005 | 0.14 ± 0.007 | 0.177 ± 0.013 | 0.172 ± 0.023 |

*RMSE values (mean ± standard error) for each Immediate Geographic Region under five predictive configurations. The table compares the baseline SARIMAX approach (hospitalization-only) against four LSTM-based models incorporating different combinations of hospitalization time series, clinical-search activity, and climatic predictors. Results are computed from triplicate model runs and highlight geographic variability in prediction accuracy.*
