# Supplementary material for: Dengue hospitalizations in Brazil: Forecasting with climatic and physicians’ digital search data under real-world reporting delays
Source: PLOS Digit Health. 2026 May 29;5(5):e0001206. doi: 10.1371/journal.pdig.0001206 (PMC13221015; doi:10.1371/journal.pdig.0001206)
Supplement: S5 Table — Pairwise statistical tests comparing predictive performance. (DOCX) [file pdig.0001206.s005.docx]

**S5 Table. t-statistics and p-values for pairwise comparisons between LSTM model configurations under Ideal-data scenarios**

| Immediate Geographic Region | Comparison between models | t-statistic | p-value |
| --- | --- | --- | --- |
| Alegre | Hospitalization vs Hospitalization + Climate | 15.423 | 0.004 |
| Alegre | Hospitalization vs Hospitalization + Clinical search + Climate | 5.246 | 0.034 |
| Belo Horizonte | Hospitalization vs Hospitalization + Clinical search | -41.708 | 0.001 |
| Belo Horizonte | Hospitalization vs Hospitalization + Clinical search + Climate | -19.661 | 0.003 |
| Campina Grande | Hospitalization vs Hospitalization + Climate | 23.77 | 0.002 |
| Campina Grande | Hospitalization vs Hospitalization + Clinical search | -4.438 | 0.047 |
| Campina Grande | Hospitalization vs Hospitalization + Clinical search + Climate | 26.085 | 0.001 |
| Campos dos Goytacazes | Hospitalization vs Hospitalization + Clinical search + Climate | -7.395 | 0.018 |
| Catalão | Hospitalization vs Hospitalization + Climate | 6.861 | 0.021 |
| Distrito Federal | Hospitalization vs Hospitalization + Clinical search + Climate | -28.559 | 0.001 |
| Distrito Federal | Hospitalization vs Hospitalization + Clinical search | -33.589 | 0.001 |
| Frederico Westphalen | Hospitalization vs Hospitalization + Climate | 8.738 | 0.013 |
| Frederico Westphalen | Hospitalization vs Hospitalization + Clinical search + Climate | 14.652 | 0.005 |
| Ijuí | Hospitalization vs Hospitalization + Climate | 4.866 | 0.04 |
| Juiz de Fora | Hospitalization vs Hospitalization + Clinical search + Climate | 69.955 | <0.001 |
| Linhares | Hospitalization vs Hospitalization + Clinical search | 6.848 | 0.021 |
| Maringá | Hospitalization vs Hospitalization + Clinical search + Climate | 5.863 | 0.028 |
| Marília | Hospitalization vs Hospitalization + Clinical search + Climate | 10.185 | 0.01 |
| Marília | Hospitalization vs Hospitalization + Climate | 8.032 | 0.015 |
| Oliveira | Hospitalization vs Hospitalization + Clinical search | -44.854 | <0.001 |
| Oliveira | Hospitalization vs Hospitalization + Clinical search + Climate | -19.603 | 0.003 |
| Passo Fundo | Hospitalization vs Hospitalization + Clinical search + Climate | 9.105 | 0.012 |
| Passo Fundo | Hospitalization vs Hospitalization + Climate | 16.274 | 0.004 |
| Passos | Hospitalization vs Hospitalization + Clinical search | -10.118 | 0.01 |
| Passos | Hospitalization vs Hospitalization + Clinical search + Climate | -12.498 | 0.006 |
| Pirapora | Hospitalization vs Hospitalization + Clinical search + Climate | 9.152 | 0.012 |
| Pirapora | Hospitalization vs Hospitalization + Climate | 8.371 | 0.014 |
| Porto Alegre | Hospitalization vs Hospitalization + Clinical search | -4.799 | 0.041 |
| Ribeirão Preto | Hospitalization vs Hospitalization + Climate | -11.476 | 0.008 |
| Rio de Janeiro | Hospitalization vs Hospitalization + Climate | -6.744 | 0.021 |
| Salvador | Hospitalization vs Hospitalization + Clinical search + Climate | -6.617 | 0.022 |
| Salvador | Hospitalization vs Hospitalization + Climate | -8.921 | 0.012 |
| Santa Cruz do Sul | Hospitalization vs Hospitalization + Clinical search | -67.705 | <0.001 |
| Santa Cruz do Sul | Hospitalization vs Hospitalization + Clinical search + Climate | -6.346 | 0.024 |
| Santa Maria | Hospitalization vs Hospitalization + Climate | 5.494 | 0.032 |
| São Miguel do Oeste | Hospitalization vs Hospitalization + Clinical search + Climate | 23.227 | 0.002 |
| São Miguel do Oeste | Hospitalization vs Hospitalization + Climate | 21.231 | 0.002 |
| São Paulo | Hospitalization vs Hospitalization + Climate | -20.768 | 0.002 |
| São Paulo | Hospitalization vs Hospitalization + Clinical search | -19.914 | 0.003 |
| São Paulo | Hospitalization vs Hospitalization + Clinical search + Climate | -15.111 | 0.004 |
| Uberaba | Hospitalization vs Hospitalization + Climate | 4.539 | 0.045 |
| Uberlândia | Hospitalization vs Hospitalization + Climate | -8.454 | 0.014 |

*Pairwise comparisons between the baseline Hospitalization-only LSTM model and three enhanced configurations: (i) Hospitalization + Clinical search, (ii) Hospitalization + Climate, and (iii) Hospitalization + Clinical search + Climate. For each IGR, RMSE values from triplicate runs were compared using a paired t-test (Shapiro–Wilk p > 0.05) or Wilcoxon signed-rank test otherwise. Statistical significance was defined as p < 0.05. Positive t-statistics indicate improved performance of the enhanced model (lower RMSE), whereas negative values indicate worse performance relative to the baseline.*
