# Supplementary material for: Dengue hospitalizations in Brazil: Forecasting with climatic and physicians’ digital search data under real-world reporting delays
Source: PLOS Digit Health. 2026 May 29;5(5):e0001206. doi: 10.1371/journal.pdig.0001206 (PMC13221015; doi:10.1371/journal.pdig.0001206)
Supplement: S6 Table — Root mean squared error values across regions accounting for reporting delays. (DOCX) [file pdig.0001206.s006.docx]

**S6 Table. Predictive performance (RMSE ± standard error) of all modeling configurations under the Real-world-data scenario**

| Immediate Geographic Region | Sarima Univariate | Hospitalization | Hospitalization + Clinical search | Hospitalization + Climate | Hospitalization + Clinical search + Climate |
| --- | --- | --- | --- | --- | --- |
| Alegre | 0.183 ± 0.0 | 0.232 ± 0.012 | 0.181 ± 0.011 | 0.158 ± 0.008 | 0.184 ± 0.009 |
| Belo Horizonte | 0.341 ± 0.0 | 0.36 ± 0.006 | 0.316 ± 0.003 | 0.308 ± 0.004 | 0.325 ± 0.003 |
| Campina Grande | 0.21 ± 0.0 | 0.208 ± 0.005 | 0.165 ± 0.002 | 0.127 ± 0.001 | 0.138 ± 0.004 |
| Campos dos Goytacazes | 0.313 ± 0.0 | 0.363 ± 0.001 | 0.31 ± 0.002 | 0.338 ± 0.006 | 0.344 ± 0.003 |
| Catalão | 0.194 ± 0.0 | 0.184 ± 0.002 | 0.161 ± 0.004 | 0.153 ± 0.002 | 0.146 ± 0.001 |
| Cruz Alta | 0.139 ± 0.0 | 0.191 ± 0.007 | 0.155 ± 0.012 | 0.153 ± 0.022 | 0.152 ± 0.012 |
| Distrito Federal | 0.324 ± 0.0 | 0.317 ± 0.015 | 0.289 ± 0.004 | 0.322 ± 0.018 | 0.296 ± 0.006 |
| Frederico Westphalen | 0.182 ± 0.0 | 0.253 ± 0.022 | 0.161 ± 0.012 | 0.193 ± 0.004 | 0.153 ± 0.015 |
| Ijuí | 0.246 ± 0.0 | 0.285 ± 0.004 | 0.219 ± 0.009 | 0.237 ± 0.019 | 0.22 ± 0.004 |
| Juiz de Fora | 0.326 ± 0.0 | 0.35 ± 0.002 | 0.249 ± 0.005 | 0.262 ± 0.01 | 0.227 ± 0.024 |
| Linhares | 0.19 ± 0.0 | 0.229 ± 0.016 | 0.147 ± 0.004 | 0.245 ± 0.001 | 0.21 ± 0.01 |
| Maringá | 0.206 ± 0.0 | 0.212 ± 0.024 | 0.186 ± 0.005 | 0.18 ± 0.024 | 0.16 ± 0.009 |
| Marília | 0.243 ± 0.0 | 0.23 ± 0.005 | 0.186 ± 0.01 | 0.143 ± 0.005 | 0.14 ± 0.001 |
| Oliveira | 0.24 ± 0.0 | 0.267 ± 0.003 | 0.247 ± 0.001 | 0.245 ± 0.005 | 0.243 ± 0.006 |
| Passo Fundo | 0.178 ± 0.0 | 0.256 ± 0.006 | 0.171 ± 0.004 | 0.164 ± 0.006 | 0.143 ± 0.008 |
| Passos | 0.181 ± 0.0 | 0.252 ± 0.004 | 0.165 ± 0.003 | 0.228 ± 0.015 | 0.178 ± 0.012 |
| Pirapora | 0.202 ± 0.0 | 0.259 ± 0.004 | 0.281 ± 0.006 | 0.174 ± 0.009 | 0.227 ± 0.025 |
| Porto Alegre | 0.222 ± 0.0 | 0.28 ± 0.009 | 0.232 ± 0.004 | 0.2 ± 0.027 | 0.214 ± 0.008 |
| Ribeirão Preto | 0.286 ± 0.0 | 0.307 ± 0.009 | 0.216 ± 0.002 | 0.293 ± 0.005 | 0.256 ± 0.016 |
| Rio de Janeiro | 0.287 ± 0.0 | 0.294 ± 0.007 | 0.215 ± 0.013 | 0.269 ± 0.012 | 0.225 ± 0.003 |
| Salvador | 0.315 ± 0.0 | 0.116 ± 0.004 | 0.097 ± 0.008 | 0.118 ± 0.001 | 0.114 ± 0.006 |
| Santa Cruz do Sul | 0.268 ± 0.0 | 0.273 ± 0.007 | 0.254 ± 0.002 | 0.256 ± 0.01 | 0.247 ± 0.001 |
| Santa Maria | 0.116 ± 0.0 | 0.201 ± 0.013 | 0.141 ± 0.007 | 0.126 ± 0.007 | 0.119 ± 0.003 |
| São Miguel do Oeste | 0.114 ± 0.0 | 0.135 ± 0.001 | 0.106 ± 0.001 | 0.062 ± 0.002 | 0.06 ± 0.001 |
| São Paulo | 0.364 ± 0.0 | 0.375 ± 0.001 | 0.374 ± 0.002 | 0.372 ± 0.004 | 0.37 ± 0.003 |
| Uberaba | 0.18 ± 0.0 | 0.219 ± 0.005 | 0.141 ± 0.006 | 0.141 ± 0.002 | 0.129 ± 0.007 |
| Uberlândia | 0.2 ± 0.0 | 0.179 ± 0.012 | 0.2 ± 0.007 | 0.24 ± 0.033 | 0.214 ± 0.022 |

*RMSE values (mean ± standard error) across the 27 Immediate Geographic Regions (IGRs) for the five predictive models evaluated under the Real-world-data scenario, which incorporates reporting delays in hospitalization records. The table reports results for: (i) SARIMAX–Hospitalization, (ii) LSTM Hospitalization-only, (iii) LSTM Hospitalization + Clinical search, (iv) LSTM Hospitalization + Climate, and (v) the fully Integrated model. All LSTM estimates reflect the average of triplicate runs. Lower RMSE values indicate better predictive accuracy. These results complement the distributions illustrated in Figure 5.*
