# Supplementary material for: Dengue hospitalizations in Brazil: Forecasting with climatic and physicians’ digital search data under real-world reporting delays
Source: PLOS Digit Health. 2026 May 29;5(5):e0001206. doi: 10.1371/journal.pdig.0001206 (PMC13221015; doi:10.1371/journal.pdig.0001206)
Supplement: S7 Table — Pairwise statistical tests comparing predictive performance under reporting delays. (DOCX) [file pdig.0001206.s007.docx]

**S7 Table. Pairwise statistical comparisons between predictive models under the Real-world-data scenario**

| Immediate Geographic Region | Comparison between models | t-statistic | p-value |
| --- | --- | --- | --- |
| Alegre | Hospitalization vs Hospitalization + Climate | 20.56 | 0.002 |
| Alegre | Hospitalization vs Hospitalization + Clinical search + Climate | 5.324 | 0.034 |
| Belo Horizonte | Hospitalization vs Hospitalization + Climate | 12.184 | 0.007 |
| Belo Horizonte | Hospitalization vs Hospitalization + Clinical search + Climate | 7.725 | 0.016 |
| Belo Horizonte | Hospitalization vs Hospitalization + Clinical search | 14.73 | 0.005 |
| Campina Grande | Hospitalization vs Hospitalization + Clinical search + Climate | 13.841 | 0.005 |
| Campina Grande | Hospitalization vs Hospitalization + Climate | 27.722 | 0.001 |
| Campina Grande | Hospitalization vs Hospitalization + Clinical search | 23.779 | 0.002 |
| Campos dos Goytacazes | Hospitalization vs Hospitalization + Climate | 6.278 | 0.024 |
| Campos dos Goytacazes | Hospitalization vs Hospitalization + Clinical search + Climate | 9.096 | 0.012 |
| Campos dos Goytacazes | Hospitalization vs Hospitalization + Clinical search | 28.541 | 0.001 |
| Catalão | Hospitalization vs Hospitalization + Clinical search | 9.056 | 0.012 |
| Catalão | Hospitalization vs Hospitalization + Climate | 58.538 | <0.001 |
| Catalão | Hospitalization vs Hospitalization + Clinical search + Climate | 24.029 | 0.002 |
| Cruz Alta | Hospitalization vs Hospitalization + Clinical search + Climate | 4.757 | 0.041 |
| Cruz Alta | Hospitalization vs Hospitalization + Clinical search | 9.413 | 0.011 |
| Frederico Westphalen | Hospitalization vs Hospitalization + Clinical search | 9.757 | 0.01 |
| Frederico Westphalen | Hospitalization vs Hospitalization + Clinical search + Climate | 7.406 | 0.018 |
| Frederico Westphalen | Hospitalization vs Hospitalization + Climate | 5.758 | 0.029 |
| Ijuí | Hospitalization vs Hospitalization + Clinical search + Climate | 16.772 | 0.004 |
| Ijuí | Hospitalization vs Hospitalization + Clinical search | 18.11 | 0.003 |
| Juiz de Fora | Hospitalization vs Hospitalization + Clinical search | 48.819 | <0.001 |
| Juiz de Fora | Hospitalization vs Hospitalization + Clinical search + Climate | 8.122 | 0.015 |
| Marília | Hospitalization vs Hospitalization + Climate | 16.203 | 0.004 |
| Marília | Hospitalization vs Hospitalization + Clinical search | 5.236 | 0.035 |
| Marília | Hospitalization vs Hospitalization + Clinical search + Climate | 29.95 | 0.001 |
| Oliveira | Hospitalization vs Hospitalization + Clinical search + Climate | 4.678 | 0.043 |
| Oliveira | Hospitalization vs Hospitalization + Climate | 5.35 | 0.033 |
| Oliveira | Hospitalization vs Hospitalization + Clinical search | 9.043 | 0.012 |
| Passo Fundo | Hospitalization vs Hospitalization + Clinical search | 36.044 | 0.001 |
| Passo Fundo | Hospitalization vs Hospitalization + Clinical search + Climate | 28.497 | 0.001 |
| Passos | Hospitalization vs Hospitalization + Clinical search + Climate | 10.852 | 0.008 |
| Passos | Hospitalization vs Hospitalization + Clinical search | 29.749 | 0.001 |
| Pirapora | Hospitalization vs Hospitalization + Clinical search | -4.493 | 0.046 |
| Pirapora | Hospitalization vs Hospitalization + Climate | 15.45 | 0.004 |
| Porto Alegre | Hospitalization vs Hospitalization + Clinical search | 8.993 | 0.012 |
| Porto Alegre | Hospitalization vs Hospitalization + Clinical search + Climate | 61.631 | <0.001 |
| Porto Alegre | Hospitalization vs Hospitalization + Climate | 7.952 | 0.015 |
| Ribeirão Preto | Hospitalization vs Hospitalization + Clinical search | 18.815 | 0.003 |
| Rio de Janeiro | Hospitalization vs Hospitalization + Clinical search | 11.873 | 0.007 |
| Rio de Janeiro | Hospitalization vs Hospitalization + Clinical search + Climate | 21.583 | 0.002 |
| Salvador | Hospitalization vs Hospitalization + Clinical search | 10.326 | 0.009 |
| Santa Cruz do Sul | Hospitalization vs Hospitalization + Clinical search + Climate | 6.217 | 0.025 |
| Santa Cruz do Sul | Hospitalization vs Hospitalization + Clinical search | 5.682 | 0.03 |
| Santa Maria | Hospitalization vs Hospitalization + Clinical search + Climate | 9.093 | 0.012 |
| Santa Maria | Hospitalization vs Hospitalization + Clinical search | 17.854 | 0.003 |
| Santa Maria | Hospitalization vs Hospitalization + Climate | 15.143 | 0.004 |
| São Miguel do Oeste | Hospitalization vs Hospitalization + Clinical search + Climate | 54.346 | <0.001 |
| São Miguel do Oeste | Hospitalization vs Hospitalization + Climate | 101.508 | <0.001 |
| Uberaba | Hospitalization vs Hospitalization + Clinical search | 30.409 | 0.001 |
| Uberaba | Hospitalization vs Hospitalization + Climate | 23.519 | 0.002 |
| Uberaba | Hospitalization vs Hospitalization + Clinical search + Climate | 13.122 | 0.006 |

*Pairwise comparisons between the baseline Hospitalization-only LSTM model and three enhanced configurations: (i) Hospitalization + Clinical search, (ii) Hospitalization + Climate, and (iii) Hospitalization + Clinical search + Climate, under the Real-world-data scenario, which incorporates delays in hospitalization reporting. For each IGR, RMSE values from triplicate runs were compared using a paired t-test (Shapiro–Wilk p > 0.05) or Wilcoxon signed-rank test otherwise. Statistical significance was defined as p < 0.05. Positive t-statistics indicate improved performance of the enhanced model (lower RMSE), whereas negative values indicate worse performance relative to the baseline..*
