# Supplementary figures and images for: Dengue hospitalizations in Brazil: Forecasting with climatic and physicians’ digital search data under real-world reporting delays
Source: PLOS Digit Health. 2026 May 29;5(5):e0001206. doi: 10.1371/journal.pdig.0001206 (PMC13221015; doi:10.1371/journal.pdig.0001206)

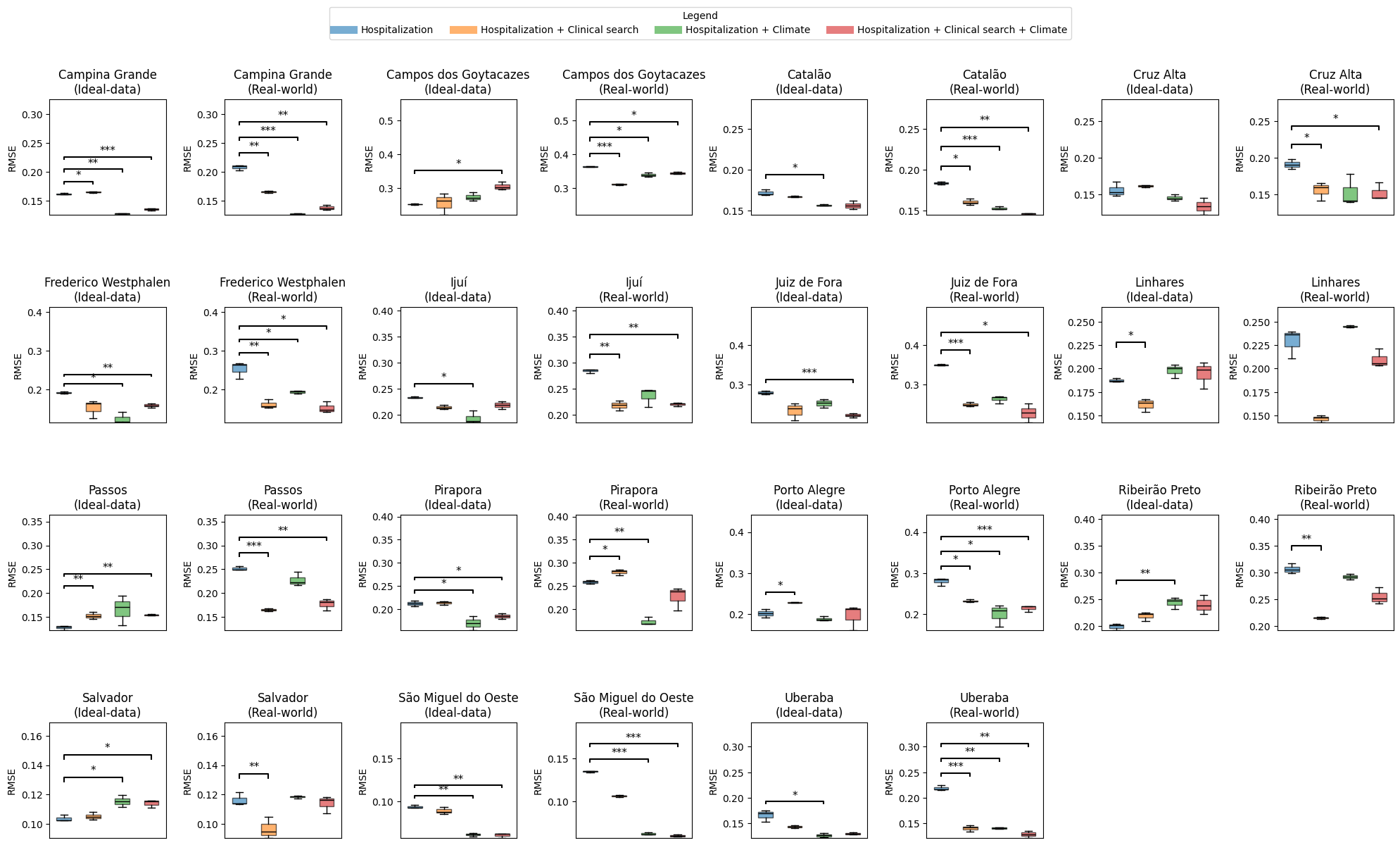

Supplement: S1 Fig — (TIF) [file pdig.0001206.s010.tif]

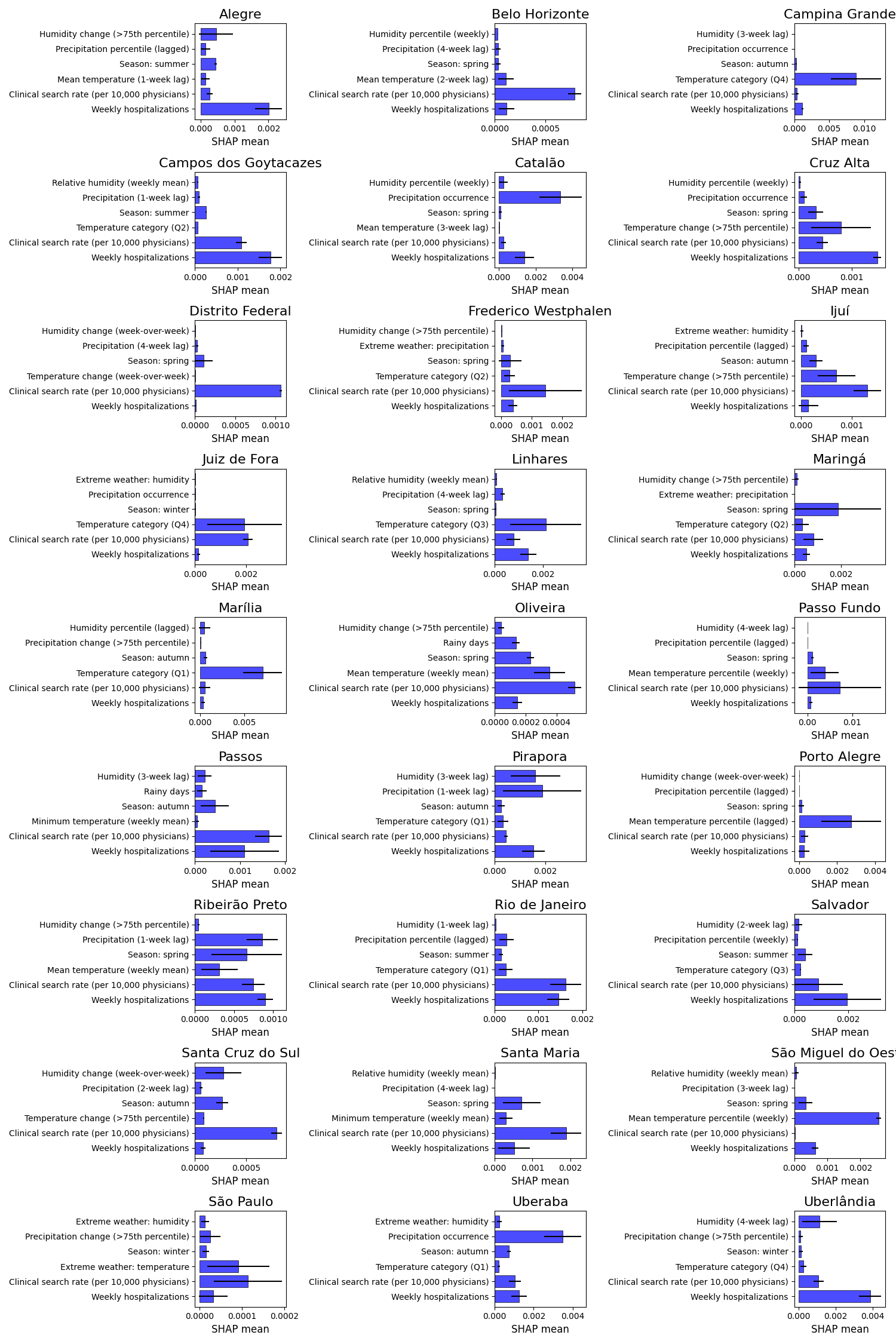

Supplement: S2 Fig — (TIF) [file pdig.0001206.s011.tif]

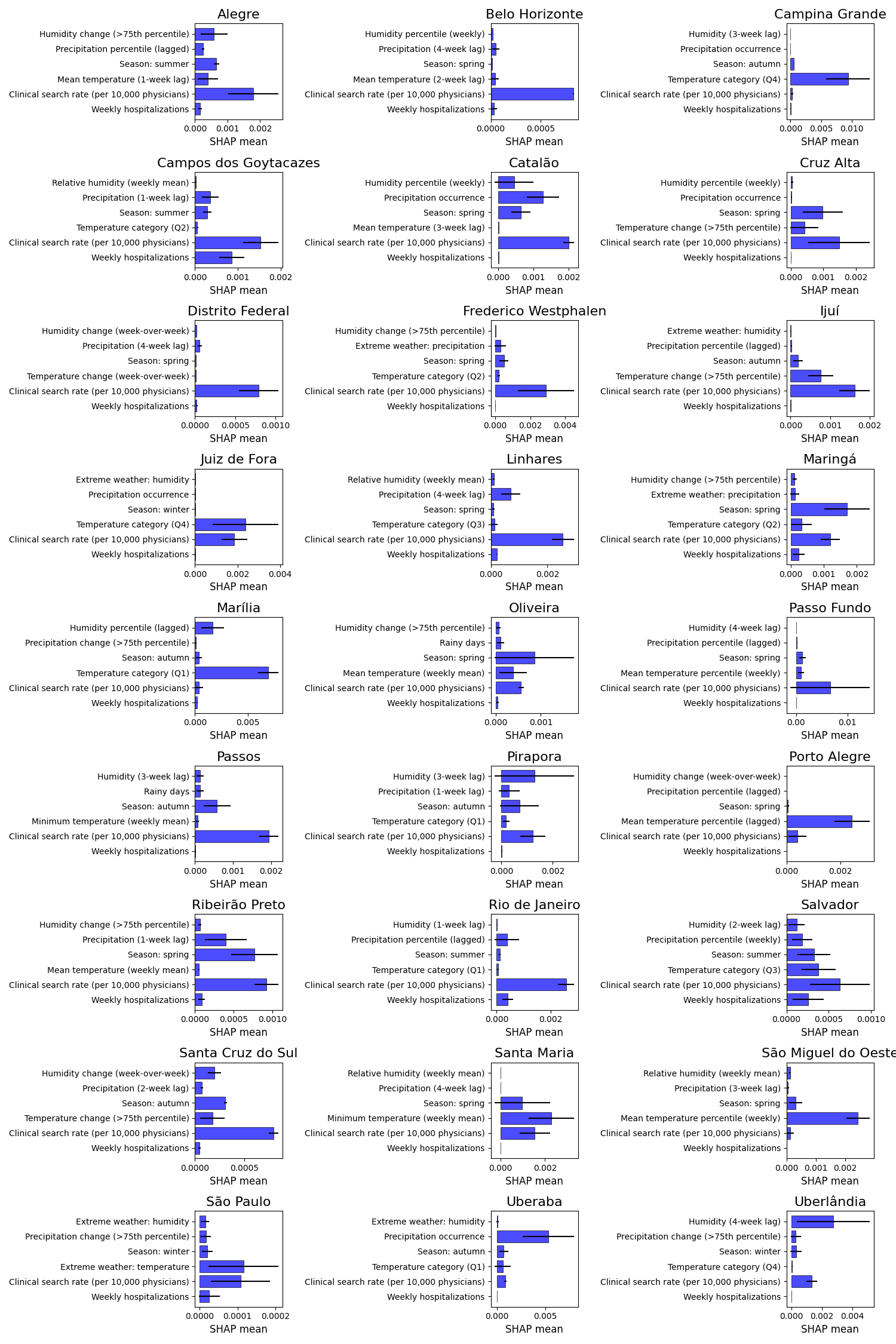

Supplement: S3 Fig — (TIF) [file pdig.0001206.s012.tif]
